# Development of an In Vitro Model of SARS-CoV-Induced Acute Lung Injury for Studying New Therapeutic Approaches

**DOI:** 10.3390/antiox11101910

**Published:** 2022-09-27

**Authors:** Yulia A. Shevtsova, Kirill V. Goryunov, Valentina A. Babenko, Irina B. Pevzner, Valentina V. Vtorushina, Evgeniya V. Inviyaeva, Lyubov V. Krechetova, Ljubava D. Zorova, Egor Y. Plotnikov, Dmitry B. Zorov, Gennady T. Sukhikh, Denis N. Silachev

**Affiliations:** 1 V.I. Kulakov National Medical Research Center of Obstetrics, Gynecology and Perinatology, 117997 Moscow, Russia; 2 Faculty of Bioengineering and Bioinformatics, Lomonosov Moscow State University, 119992 Moscow, Russia; 3 A.N. Belozersky Institute of Physico-Chemical Biology, Lomonosov Moscow State University, 119992 Moscow, Russia

**Keywords:** COVID-19, cytokine storm, model, lung epithelium, oxidative stress

## Abstract

One of the causes of death of patients infected by SARS-CoV-2 is the induced respiratory failure caused by excessive activation of the immune system, the so-called “cytokine storm”, leading to damage to lung tissue. In vitro models reproducing various stages of the disease can be used to explore the pathogenetic mechanisms and therapeutic approaches to treating the consequences of a cytokine storm. We have developed an in vitro test system for simulating damage to the pulmonary epithelium as a result of the development of a hyperinflammatory reaction based on the co-cultivation of pulmonary epithelial cells (A549 cells) and human peripheral blood mononuclear cells (PBMC) primed with lipopolysaccharide (LPS). In this model, after 24 h of co-cultivation, a sharp decrease in the rate of proliferation of A549 cells associated with the intrinsic development of oxidative stress and, ultimately, with the induction of PANoptotic death were observed. There was a significant increase in the concentration of 40 cytokines/chemokines in a conditioned medium, including TNF-α, IFN-α, IL-6, and IL-1a, which corresponded to the cytokine profile in patients with severe manifestation of COVID-19. In order to verify the model, the analysis of the anti-inflammatory effects of well-known substances (dexamethasone, LPS from Rhodobacter sphaeroides (LPS-RS), polymyxin B), as well as multipotent mesenchymal stem cells (MSC) and MSC-derived extracellular vesicles (EVs) was carried out. Dexamethasone and polymyxin B restored the proliferative activity of A549 cells and reduced the concentration of proinflammatory cytokines. MSC demonstrated an ambivalent effect through stimulated production of both pro-inflammatory cytokines and growth factors that regenerate lung tissue. LPS-RS and EVs showed no significant effect. The developed test system can be used to study molecular and cellular pathological processes and to evaluate the effectiveness of various therapeutic approaches for the correction of hyperinflammatory response in COVID-19 patients.

## 1. Introduction

Since the first outburst of cases of severe acute respiratory syndrome (SARS) in 2002 caused by SARS-associated coronavirus (SARS-CoV), much efforts were made to unravel the mechanisms of SARS-CoV-induced lung tissue injury and develop new options for effective treatment intended primarily to prevent hyperactivation of the immune system. The next coronavirus disease pandemic outburst happened in 2019 (COVID-19), caused by SARS-CoV-2, and proved the need to accelerate research in this area. It is obvious that appropriate experimental models reproducing COVID-19 in humans are required. Clinical data indicate that COVID-19 progression could be different: from asymptomatic to severe (e.g., associated with dyspnea, hypoxia, or >50 percent lung damage) or critical (e.g., associated with respiratory failure, shock, or multiple organ failure) [1,2,3]. The immune response to viral infection with the risk of cytokine storm plays a crucial role in the severity of the disease [4]. In particular, the result of cytokine storm is the damage to the lung tissue and progressive respiratory failure, which, in critical cases, can cause the death of the patient [5]. Considering cytokine storm as one of the main causes of a poor outcome, working experimental models are highly needed to reproduce this process.

Basically, all existing in vivo models of viral pneumonia have significant limitations. For example, SARS-CoV infection in mice is manifested by a rapid immune response and elimination of the virus, thus excluding the development of a cytokine storm [6,7]. On the other hand, the use of a mouse-adapted SARS-coronavirus caused the mortality of mice without massive infiltration of immune cells into the lungs due to rapid amplification of the virus and severe viremia [8]. Among limitations of the developed in vivo models are the complexity of manipulations with animals, limitations in the use of the infectious agent, and the inadequacy of the clinical picture due to differences in the immune systems of humans and animals. It is worth noting that the developed models did not reproduce the severe progression of the disease with cytotoxic damage to the lung tissue. All this hinders both the development of pathogenetic therapy aimed at correcting immune response and the research of disease mechanisms specific for humans. Moreover, in vivo models do not provide a platform for extensive drug screening, since the use of a large number of animals requires significant resources for manipulations and maintenance and contradicts with 3R principles.

In vitro models are widely accepted for screening drug candidates prior to in vivo preclinical studies [9]. In vitro models can provide information about the role of immune cells in the inflammatory response, help to define the spectrum of factors secreted by immune cells, and examine the mechanisms of intercellular communication and immune reactions resembling inflammation in lung tissue during viral pneumonia. In vitro models allow the use of human cells, i.e., cell lines or primary cells from donors, to explore physiological aspects of human biology. Thus, we assume that the development of an in vitro model that adequately mimics the pathological processes of lung epithelial damage under cytokine storm can resolve several fundamental issues in SARS therapy. For these purposes, it looks reasonable to use complex cell systems, consisting of human lung epithelial cells (cell lines) and immune cells (peripheral blood mononuclear cells) from donors. Instead of an infectious agent, we propose to use bacterial lipopolysaccharide (LPS) to trigger an immune response. LPS is an agonist of toll-like receptor 4 (TLR4), and it activates innate immune reactions [10]. To test therapeutics, an in vitro model should be easily reproducible and consisting of readily available cellular components coupled with the system for monitoring of cell proliferation and cell death, such as the iCELLigence System [11].

Moreover, in vitro models make it possible to apply a wider range of approaches to the study of molecular and cellular pathogenetic mechanisms of inflammation-mediated lung injury. Thus, it is known that cytokine storm also causes the development of oxidative stress due to the production of reactive oxygen species (ROS) by the immune cells [12], but mechanisms of ROS production in lung epithelial cells under conditions of a cytotoxic storm are not clearly described due to methodological difficulties in such studies in vivo. We suggested that mitochondria may be one of the sources of ROS in these pathological conditions that could be examined using cell culture models.

To date, there are several proposed approaches for SARS treatment, e.g., the use of pharmacological and biotechnological products, as well as cell therapy [13,14], but the efficacy of those approaches has not yet been fully established. There is a limited spectrum of therapeutic agents recommended by WHO, e.g., corticosteroids, interleukin-6 (IL-6) receptor blockers or janus kinase (JAK) inhibitors, designed to suppress immune hyperactivation [15,16]. There are a large number of therapeutic drugs under development at preclinical stages with limited data obtained on their clinical efficacy. For example, TLR4 antagonists have been shown to alleviate cytokine storm [17,18,19]. Many research teams suggest using cell-based therapies, particularly based on multipotent mesenchymal stem cells (MSC) or MSC-derived extracellular vesicles (EVs), which provide a wide spectrum of positive therapeutic effects [20,21] but have not yet passed thorough clinical trials.

The aim of the study was to develop a comprehensive in vitro model reproducing damage to the lung epithelium under the cytotoxic action of cytokines and consisting of human lung epithelial cells, leukocytes, and endotoxin with further testing of the developed model using WHO-approved therapeutic agents with proven preclinical/clinical efficacy.

## 2. Materials and Methods

### 2.1. A549 Cultivation

A549 cells are alveolar basal epithelial cells, originally obtained from an explanted pulmonary carcinoma tumor of a 58-year-old Caucasian male by D. J. Giard et al. in 1972 [22]. Human lung cancer cells (A549) from the vertebrate cell culture bank of the Institute of Cytology of the Russian Academy of Sciences were cultured in Dulbecco’s Modified Eagle Medium DMEM/F12 with stabilized glutamine (Biolot, St. Petersburg, Russia), supplemented with 10% fetal bovine serum (Biosera, Nuaille, France) and 1% antibiotic–antimycotic (Gibco, Waltham, MA, USA). Cell cultures were grown in a CO_2_ incubator (Eppendorf, Hamburg, Germany) at 37 °C and 5% CO_2_.

### 2.2. Blood Sampling and Peripheral Blood Mononuclear Cells (PBMC) Isolation

The blood sampling procedure was carried out in accordance with the Helsinki Declaration of the World Medical Association and with the permission of the local Ethics Committee of the V.I. Kulakov National Medical Research Center for Obstetrics, Gynecology, and Perinatology (Protocol No. 1 of 4 February 2020), and informed consent was obtained from blood donors.

Blood samples were taken from 18 healthy donors aged 24–38 years. The donors did not receive any medications for 2 weeks prior to the start of the study.

Collected venous blood was put into test tubes with EDTA (vacuum containers) with a volume of 10 mL. The blood was diluted three-fold with phosphate-buffered saline (PBS) and carefully layered over 3 mL of Ficoll–Urografin (density 1.077 g/cm^3^; Paneko, Russia) into a 15 mL conical polypropylene tube (Corning, New York, NY, USA). After centrifugation at 400× *g* for 30 min, PBMCs were washed with PBS and centrifuged for 5 min at 400× *g*. The resultant pellet was resuspended in 2 mL DMEM/F12. After cell counting in the cell counter (Countess II FL; Thermo Fisher Scientific Inc., Waltham, MA, USA), cells were diluted to a final concentration of ~1,000,000 cells per mL.

### 2.3. Real-Time Cell Proliferation Monitoring

The kinetics of A549 cell growth was analyzed using the RTCA iCELLigence™ instrument (ACEA, San Diego, CA, USA). The method is based on the use of the electrical impedance of cell-coated electrodes [23], and can be used, in particular, to study the proliferation and death of adhesive culture cells. The iCELLigence RTCA instrument was placed in a humidified incubator at 37 °C and 5% CO_2_. A549 cells were seeded onto 16-well plates with microelectrodes for 24 h before inflammation modeling and 30 h after. After a period of 15 min during which the cells were attached to the bottom of the wells, impedance measurements were started, and data were collected from each well every 15 min during the entire experimental period. The number of cells was set as a cell index; cell growth rate was estimated in a time interval of 35–50 h for modeling a cytotoxic storm.

### 2.4. MSC Isolation and Culturing

Human postpartum placentas were obtained from healthy women after healthy vaginal delivery of full-term infants at the V.I. Kulakov National Medical Research Center for Obstetrics, Gynecology, and Perinatology. All women signed a written informed consent, and further procedures were carried out in accordance with the instructions of the ethical committee of the Center. Placental tissue was washed several times with PBS (Paneco, Moscow, Russia) then mechanically crushed into small fragments and incubated with collagenase type II (Gibco, Waltham, MA, USA) for 2 h until visible fragmentation. After incubation, sedimented cells were cultivated in DMEM/F12 with stabilized glutamine (St. Petersburg, Moscow, Russia) containing 7% fetal bovine serum (Biosera, Nuaille, France) supplemented with penicillin (100 IU/mL) and streptomycin (100 μg/mL) (Gibco, Waltham, MA, USA) in a humidified atmosphere with 5% CO_2_ at 37 °C. The incubation medium was refreshed every 3–4 days to remove nonadherent cells. MSC at the third passage were used in the experiments. Before the experiment, cell viability was assessed by trypan blue exclusion (generally >95%). MSC used in the study were positive for mesenchymal stem cell markers (CD73, CD90, CD105) and negative for hematopoietic cell markers (CD14, CD20, CD45, CD34).

### 2.5. Isolation of Extracellular Vesicles

EVs were isolated from the conditioned medium of cultivated MSC at 80–90% confluence (~30 × 10^6^ cells) on the third passage 24 h after refreshment with culture medium supplemented with fetal bovine serum exposed to ultracentrifugation to deplete EVs containing in the serum as described previously [24]. The conditioned medium (50 mL) was then exposed to sequential centrifugation procedures to remove cells and debris at 400× *g* for 10 min followed by 10,000× *g* at 4 °C for 30 min. The resultant purified supernatant containing EVs was centrifuged at 108,000× *g* for 1.5 h at 4 °C using an Avanti JXN-30 high-speed centrifuge (Beckman Coulter Inc., Fullerton, CA, USA) followed by washing the pellet with PBS and the same protocol of centrifugation. The final EVs pellet was resuspended either in 30 μL PBS for cell culture study or in 1 mL for the nanoparticle tracking analysis (NTA). Vesicle samples were stored at −80 °C. The resuspended pellet from an unconditioned culture medium that had passed all the centrifugation procedures was used as an additional control sample (blank EVs). NTA was performed as described earlier [24].

### 2.6. Inflammation Modeling In Vitro

**Figure 1:**
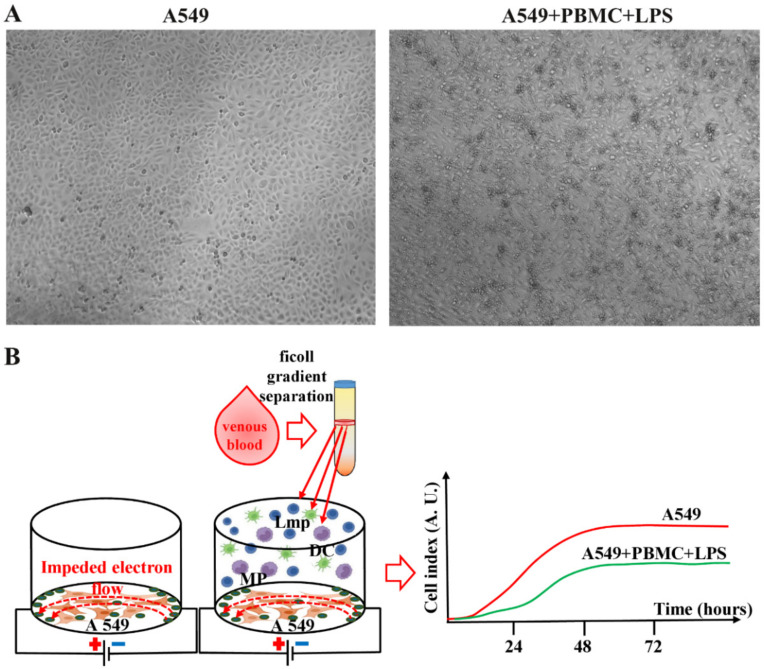
(**A**) Representative photomicrographs of untreated A549 cells and coculture A549 cells with PBMC activated by LPS after 24 h. Images acquired with 4x magnification through CELENA^®^ S Digital Imaging System (Logos Biosystems). (**B**) Experimental design. DC—dendritic cells; MP—macrophages; Lmp—lymphocytes.

A549 cells were seeded in the amount of 10,000 cells per well and cultivated for 24 h. Preliminary experiments showed that the number of A549 cells reached 16–17,000 within 24 h of cultivation. A549 cells cultivated for 1 day were cocultured with PBMC cultivated in DMEM/F12 in a ratio of 1:10 or only DMEM/F12 in a control sample (200 µL per well). Simultaneously, 5 µg/mL lipopolysaccharide (LPS; Sigma-Aldrich, St. Louis, MO, USA) solution in DMEM/F12 or DMEM/F12 alone were added to the control sample (200 µL per well) and further cultivated for 24 h (Figure 1). Then, the cocultures were treated (depending on the purpose) with: (1) dexamethasone (100 ng/mL); (2-3) inhibitors of leukocytic toll-signaling, polymyxin B (Poly B, 10 μg/mL), and LPS from *Rhodobacter sphaeroides* (LPS-RS, 5 μg/mL); (4) MSC in the amount of 10,000 cultivated on special inserts with 0.4 µm pore polyester membrane; and (5) EVs at a dose 8 × 10^8^ particles in 6 μL DMEM/F12. The conditioned medium was collected after 24 h of cocultivation, centrifugated at 2000× *g* for 5 min, and frozen at −80 °C for further cytokine analysis.

### 2.7. Cell Viability Estimation

A549 cells were collected 24 h after the modeling of inflammation and incubated with Muse Count and Viability Kit (Merck Millipore, Burlington, MA, USA) for 5 min in accordance with the manufacturer’s instructions. The number of viable cells was determined using the MUSE Cell Analyzer (Merck Millipore, Burlington, MA, USA). A total of 10,000 events were analyzed for each sample.

### 2.8. Cytokine Multiplex Assay

Samples of the conditioned medium were analyzed using the Bio-Plex Pro™ Human Cytokine Screening Kit, 48-Plex in accordance with the manufacturer’s protocol (Bio-Rad, Munich, Germany). Samples were measured in duplicates using the Bio-Plex^®^ 200 system (Bio-Rad, Munich, Germany). Concentrations of the analytes were expressed in pg/mL. The standard curve ranges on average from 0.15 pg/mL up to 3000 pg/mL. Cytokine concentrations were determined using Bio-Plex^®^ Manager Version 6.0 (Bio-Rad, Munich, Germany).

### 2.9. ROS and Lipid Peroxidation Detection

The mitochondrial ROS-sensitive fluorescent probe MitoSOX^TM^ Red (final concentration 0.9 µM) (Molecular Probes, Inc., Eugene, OR, USA) and cellular lipid reactive oxygen species fluorescent probe Image-iT™ Lipid Peroxidation Kit (C10445, ThermoFisher Scientific, Waltham, MA, USA) dissolved in DMEM/F12 without bicarbonate (final concentration 10 µM) were added to A549 cells (500 µL per well of 24-well plate) and incubated for 30 min at 37 °C, followed by a wash with DMEM/F12 without bicarbonate. A549 cells were collected after exposure to Trypsin-EDTA 0.25% solution with Hanks’ salts (Paneco LTD, Moscow, Russia). Cells were washed 3 times with pre-warmed PBS, and a fluorescence shift from 590 nm to 510 nm (for Image-iT™ Lipid Peroxidation Kit, ThermoFisher Scientific, Waltham, MA, USA) and 590 nm (for MitoSOX^TM^ Red) was measured with the S3e™ Cell Sorter (Bio-Rad). Subsequent analysis was performed using FlowJo (v10) and Python software. A minimum of 40,000 cells per sample were analyzed. Unstained A549 cells were used as controls for autofluorescence.

### 2.10. Western Blot Analysis

Wells with A549 cells after 24 h co-cultivation were washed and tripsinized, and collected cells were lysed in a sample buffer. Samples of cell lysates were loaded onto gradient (5–20%) Tris-glycine polyacrylamide gels (10 µg of total protein per lane). After electrophoresis, the gels were blotted onto PVDF membranes (Amersham Pharmacia Biotech, Newcastle, UK). The membranes were blocked with 5% (*w*/*v*) non-fat milk (SERVA, Germany) in PBS with 0.05% (*v*/*v*) Tween 20 (Panreac, Barcelona, Spain) and subsequently incubated with primary antibodies to target proteins CASP8 (PAA853Hu02, 1:1000; Cloud-Clone Corp., Wuhan, China), Phospho-MLKL (AF7419, 1:1000; Cloud-Clone Corp., Wuhan, Hubei, China), MLKL (AF7919, 1:1000; Cloud-Clone Corp., Wuhan, China), RIPK1 (PAE640Hu01, 1:1000; Cloud-Clone Corp., Wuhan, China), RIPK3 (DF10141, 1:1000; Cloud-Clone Corp., Wuhan, China), and β-actin (A1978, 1:2000; Sigma-Aldrich, St. Louis, MO, USA) as a normalization control. The membranes were incubated with secondary antibodies conjugated with horseradish peroxidase 1:10,000 (Imtek, Moscow, Russia). Detection was performed by the ChemiDoc™ MP imaging system (Bio-Rad, Hercules, CA, USA) using the WesternBright™ Enhanced Chemiluminescence kit (Advansta, Menlo Park, CA, USA).

### 2.11. Statistics

Data were tabulated and statistical analysis and graphs were generated with MS Excel 2010 (Microsoft Corporation Redmond, Washington, DC, USA) and Prism GraphPad 7 (GraphPad Software, San Diego, CA, USA). The normality of the distribution was assessed by Shapiro–Wilk test. All values are given as the mean ± standard error (SEM). Statistical analysis was performed using a one-way ANOVA followed by a Tukey’s post hoc test. For two groups experiments an unpaired Welch-corrected *t*-test was performed. Differences with a probability (*p*) value less than 0.05 were considered statistically significant.

## 3. Results

### 3.1. Inflammation Modeling

Our primary goal was to develop model conditions for cytotoxic damage to the pulmonary epithelium. As a cellular model with the characteristics of the pulmonary epithelium, we chose the A549 lung carcinoma cell line, which is a generally recognized model system for studying the function of type II alveolar cells [25]. A549 cells were seeded in an amount of 10,000 cells so that they had time to attach to the substrate and adapt. Prior to the main experiments, the optimal ratio of A549 cells to PBMC, incubation time, and LPS concentrations for PBMC activation were determined. The best experimental conditions were estimated at a ratio of A549 cells to PBMC (activated by LPS, 5 µg/mL added 24 h after seeding A549 cells) as 1:10.

**Figure 2:**
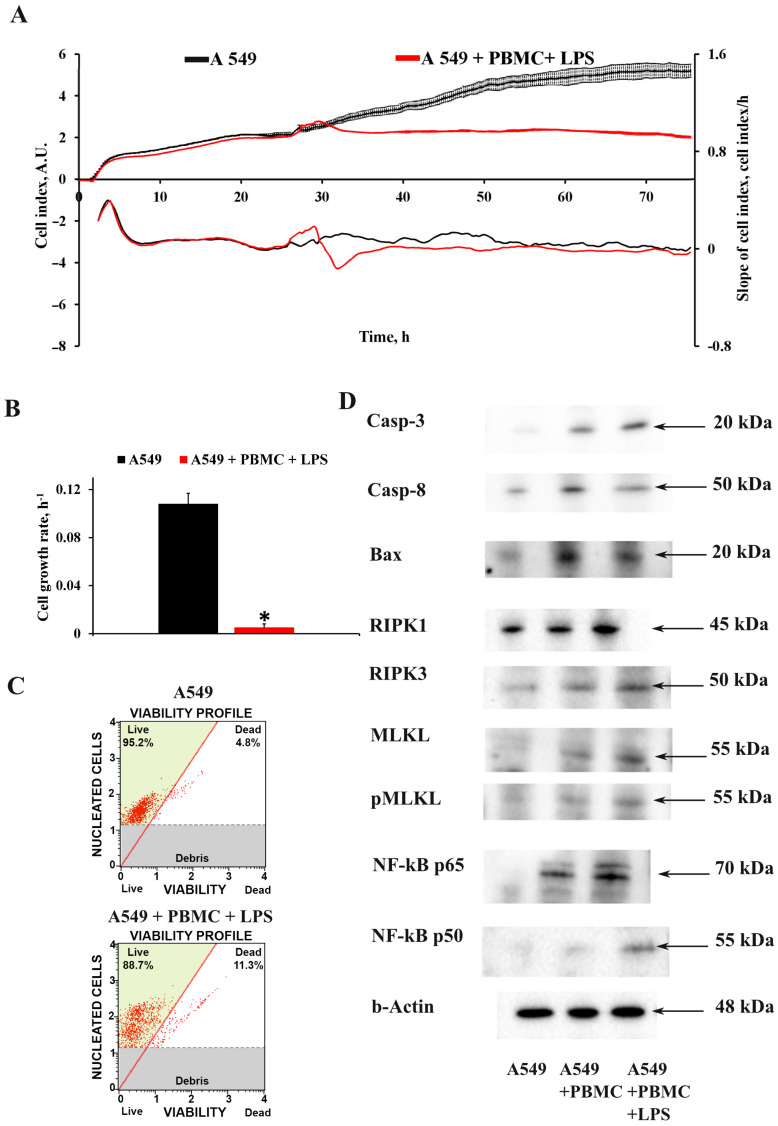
Assessment of A549 proliferation and cell death after 24-h impact of activated PBMC. (**A**) Real-time curve of cell index for A549 and (**B**) the cell growth rate measured with the RTCA iCELLigence over 55–70 h. A549 cells were seeded on the special detection plate and independence electron flow was detected and recalculated in cell index. (**C**) A549 death in the model conditions using Muse^®^ Count & Viability kit. A549 cells were collected 24 h after cocultivation with activated PBMC, and the viable cells were detected using the MUSE Cell Analyzer (Merck Millipore, Burlington, MA, USA). (**D**) Representative immunoblots for markers of PANoptosis cell death in A549. Actin staining was used as a loading control. Number of individual cultures for experiments was 3. Results are indicated as mean ± SEM; * *p* < 0.05, an unpaired Welch-corrected *t*-test was used.

The viability of intact A549 cells and those cocultured with PBMC exposed to LPS was assessed by monitoring the cell index in real time using an iCELLigence device. We found that untreated A549 cells characterized by fast proliferation exceeded the cell growth rate of A549 cells cocultured with LPS-treated PBMC by more than 10-fold (Figure 2A,B). The cell growth rate analysis measured as the slope of cell index increment showed a dramatic decrease of the growth of A549 cells after co-cultivation with LPS-treated PBMC, whereas A549 cells treated with LPS or PBMC alone showed no significant change in cell index compared to unexposed A549 cells (Figure S1).

We analyzed the effect of the proinflammatory microenvironment on the death of A549 cells. We found that the model conditions caused significant cell death; the average number of dead cells was 11.2  ±  1.2% compared to the control 4.8  ±  0.6% for A549 cells (Figure 2C.) Next, we explored the mechanism of cells death. It has been shown that the proinflammatory environment cause new recently described cell death which was called PANoptosis [26,27]. It was found that A549 cells cocultured with LPS-activated PBMC significantly increased key markers of PANoptosis. Western blot analysis revealed the main markers of the apoptotic initiator caspase-8 and the apoptotic executioner caspase-3 cleaved forms; necrosis markers RIPK1, RIPK3, and pMLKL; as well as, according to recent data [28], an overwhelming increase in the level of caspase-3 and activation of NF-kB-dependent pathway can be considered as the initiation of pyroptotic cell death, which makes it possible to judge the initiation of PANoptosis death of A549 cells.

**Figure 3:**
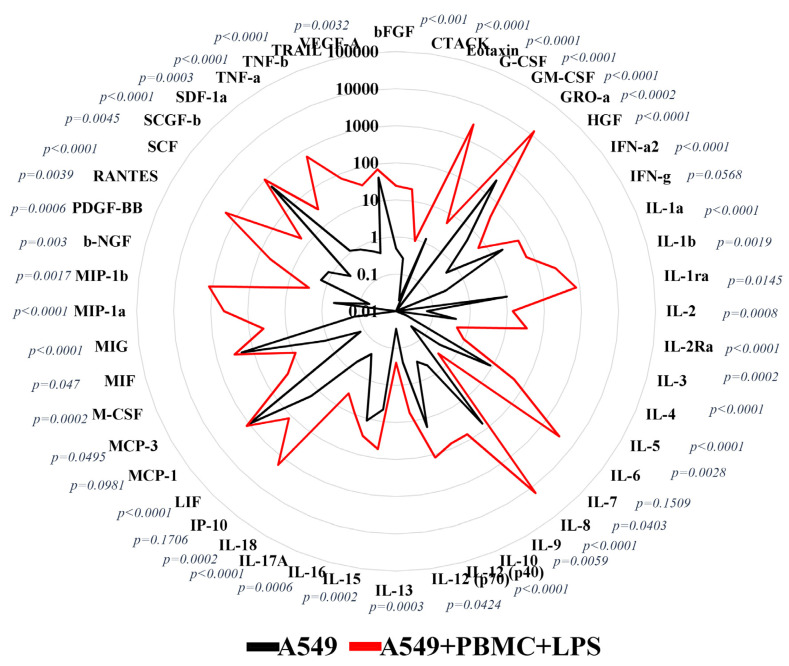
Spider plot, cytokine/chemokine concentrations (pg/mL; log scale) in conditioned medium 24 h after A549 cocultivation with PBMC primed with LPS; *n* = 5, Unpaired *t*-test with Welch’s correction.

Analysis of the cytokine profile in a conditioned medium 24 h after cocultivation of A549 cells with activated PBMC showed the formation of a pro-inflammatory microenvironment due to an increase in the concentration of cytokines and chemokines produced by leukocytes. We found a strongly elevated level in 40 of the 48 measured analytes (Figure 3), of which IFN-α (*p* < 0.0001), IL-1α (*p* < 0.0001) IL-6 (*p* = 0.0028), and TNF-α (*p* = 0.0003) should be noted as being considered more important in the initiation of a cytokine storm compared to the control conditioned medium of A549 cells [29]. The increased release of these factors in the model system was higher than in the culture medium obtained from PBMC or A549 cells treated with LPS and cultivated separately (Figure S2). Thus, the secretome obtained from the combined cultures of A549 cells and PBMC in the presence of LPS turned out to be enriched with cytokines, representative of both humoral and cell-mediated immunity, which resembles the infectious pathology of COVID-19 in humans [30].

### 3.2. Oxidative Stress in A549 Cells under Inflammation Conditions

**Figure 4:**
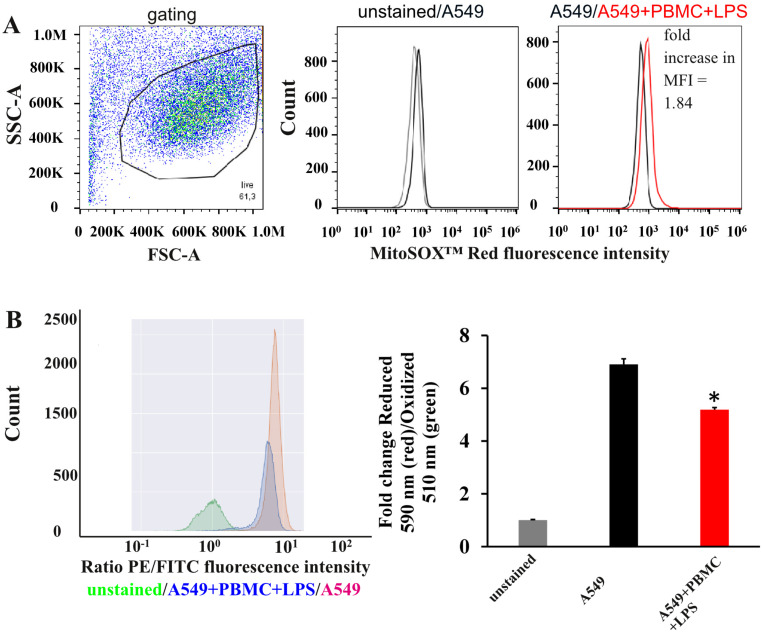
Effect of cocultivation with activated PBMC on ROS generation in A549 cells. (**A**) Evaluation of mitochondrial ROS production by MitoSOX^TM^ Red. The left figure illustrates flow-cytometric dot plots showing gating of A549 cells. The central figure shows a histogram of cytometric analysis of MitoSOX^TM^ Red on A549 cells unstained (gray) and intact A549 cells were stained with MitoSOX^TM^ Red (black). The right figure shows a histogram of cytometric analysis of MitoSOXTM Red on intact A549 cells (black) and A549 cells were collected 24 h after the modeling of inflammation (red). (**B**) Red/green fluorescence intensity ratio, indicating an increase in lipid peroxidation in A549 cells estimated by Image-iT™ Lipid Peroxidation Kit. Histograms represent the ratio of red to green in A549 cells. Data are presented as a mean  ±  SEM, n = 3. * *p* < 0.05 vs. A549 as determined by *t*-test. MFI—mean fluorescence intensity.

Next, we explored oxidative stress in A549 cells in developed model system. Using this model, we observed a statistically greater mitochondrial ROS production by about 1.8 times as measured by fluorescent signal from MitoSOX^TM^ Red, compared with untreated A549 cells (Figure 4A). To detect lipid peroxidation in our model system, we used Image-iT™ Lipid Peroxidation Kit for live cells. This kit contains specific dye C11-BODIPY(581/591), a fluorescent lipid peroxidation sensor that can freely penetrate through cell membrane and changes its emission from red to green upon peroxidation by ROS in cells [31]. The ratio of red/green fluorescence significantly decreased by 1.4 times under the conditions of co-cultivation of A549 with activated leukocytes compared with untreated A549 cells (Figure 4B).

### 3.3. The Effect of Dexamethasone on the Cytokine Profile and Cell Index of A549 Cells

**Figure 5:**
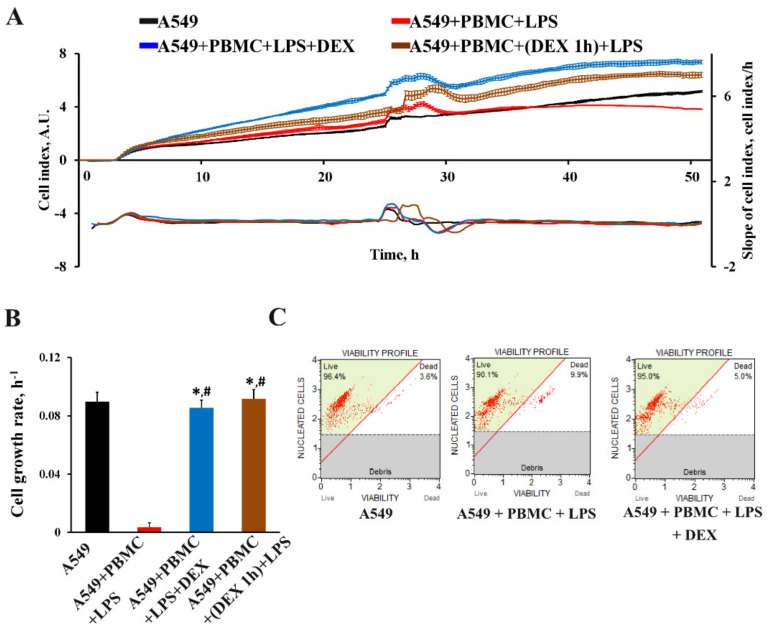
The effect of dexamethasone on the cell index and death of A549 cells within 24 h in a model system. The real-time curve of cell index for A549 cells (**A**) and quantification of the cell growth rate (**B**) measured using the RTCA iCELLigence for 35–50 h. (**C**) Cell death of A549 cells estimated using the Muse^®^ Count & Viability kit. A549 cells were collected 24 h after cocultivation with activated PBMC, and the viable cells were estimated using the MUSE Cell Analyzer (Merck Millipore, Burlington, MA, USA). The data are presented as a mean  ±  SEM, *n* = 3. * *p* < 0.05 vs. A549 + PBMC + LPS, # *p* < 0.05 vs. A549 cells as determined by one-way ANOVA with Tukey’s multiple comparisons test.

To validate our model for the applicability of drug screening, we decided to test drugs approved for the treatment of COVID-19. We chose dexamethasone as one of the most effective anti-inflammatory glucocorticosteroids, which has shown its effectiveness in the treatment of patients with COVID-19 [32,33]. In the developed in vitro model, we tested the effect of dexamethasone on the inhibition of the pro-inflammatory activity of PBMC according to two schemes. The first scheme included the simultaneous addition of dexamethasone together with leukocytes and LPS to wells with A549 cells, while the second scheme included treatment of leukocytes 1 h before cocultivation. Both modes of dexamethasone administration showed a considerably greater maximum effect on the proliferation rate of A549 cells compared to both untreated cells and those under model conditions (Figure 5A,B). The addition of dexamethasone to A549 cells simultaneously with leukocytes and LPS prevented the death of the lung epithelium (Figure 5C)

**Figure 6:**
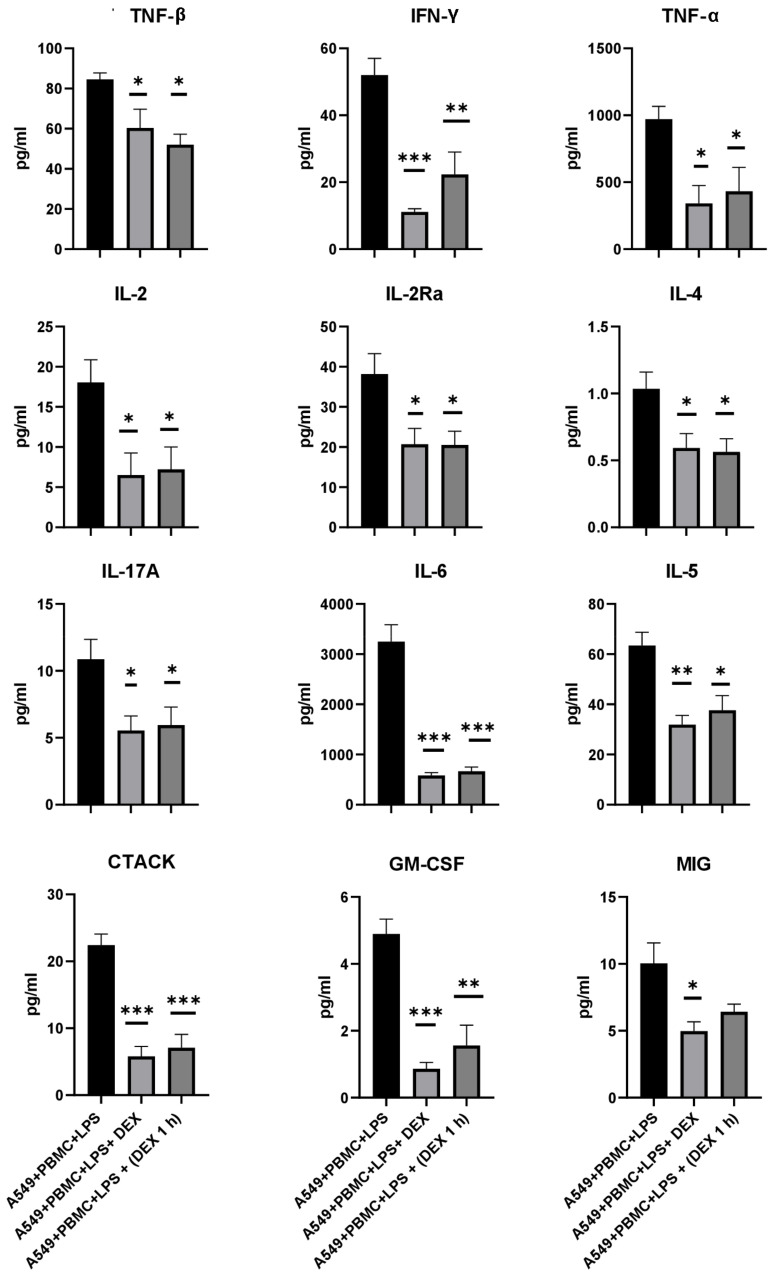
Effect of dexamethasone on cytokine/chemokine concentrations in the conditioned medium from the model system after 24 h treatment. The data are presented as a mean  ±  SEM, *n* = 3. * *p* < 0.05, ** *p* < 0.01, *** *p* < 0.001; the significance from the A549 + PBMC + LPS is assessed by a one-way ANOVA with the Tukey’s multiple comparisons test.

We evaluated the effect of dexamethasone treatment on changes in the cytokine profile in a conditioned medium after 24 h of cytokine storm simulation. A significant decrease in the concentration of 26 pro-inflammatory chemokines and cytokines in comparison with the control A549 cell culture was found regardless of the dexamethasone treatment regimen (Figure 6 and Figure S3). As shown in Figure 6, the levels of the main inflammatory cytokines TNF-β, TNF-α, and IFN-γ decreased by 1.5–2 times. A twofold decrease in the concentration of cytokines such as IL-4, IL-2, and its receptor IL-2Ra may indicate that the activation of T-cells and B-cells was inhibited by dexamethasone. We also found that the levels of IL-17A, IL-6, and IL-5 were significantly reduced by 2, 4, and 2 times, respectively. In addition, chemokines such as CTACK and MIG were significantly reduced by half (Figure 6).

### 3.4. Effect of TLR4 Inhibitors on Cytokine/Chemokine Concentrations and Proliferation of A549 Cells in the Model System

**Figure 7:**
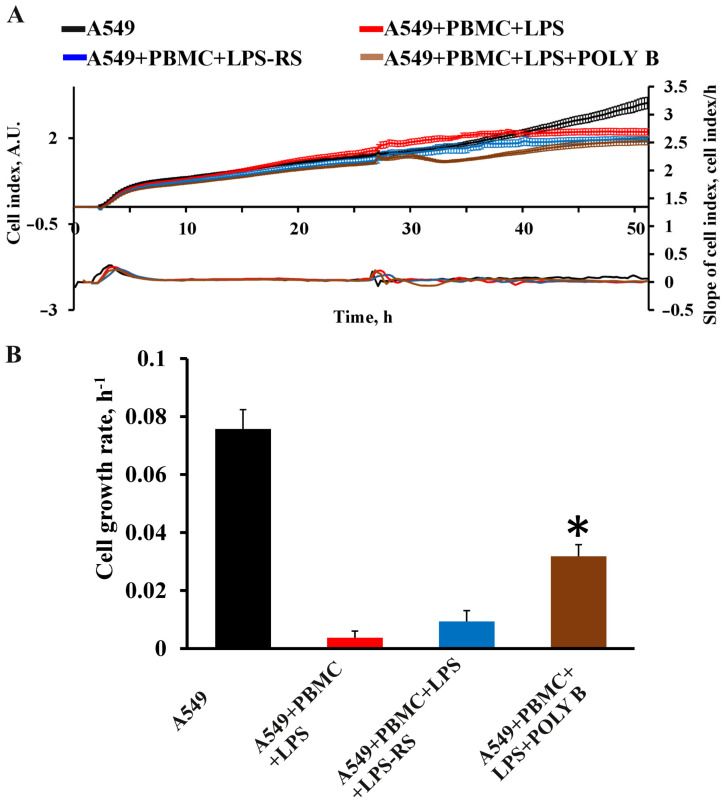
Effect of TLR4 inhibitors on proliferation of A549 cells during 24 h in model system. Real-time curve of cell index for A549 cells (**A**) and quantification of the cell growth rate (**B**) measured with the RTCA iCELLigence over 40–50 h. Data are presented as a mean  ±  SEM, *n* = 3. * *p* < 0.05 vs. A549 + PBMC + LPS as determined by one-way ANOVA with Tukey’s multiple comparisons test. LPS-RS—LPS from the photosynthetic bacterium *Rhodobacter sphaeroides*; POLY B—polymyxin B.

An additional element of the verification of the developed test system was the evaluation of the effectiveness of various therapeutic approaches according to the main criteria of effectiveness, such as the proliferation of A549 cells, as well as a change in the pro-inflammatory pattern of cytokines in the direction of its decrease. TLR4 antagonists have previously been shown to be promising drugs preventing the development of cytokine storm in patients with COVID-19 [18]. We selected well-known TLR4 antagonists such as LPS-RS and polymyxin B. LPS-RS (5 μg/mL) did not significantly affect the change in the cellular index of A549 cells (Figure 7A,B). In the group receiving polymyxin B (10 μg/mL), there was a significant increase in the cell index and proliferation rate of A549 cells compared to intact lung epithelial cells (Figure 7A,B).

**Figure 8:**
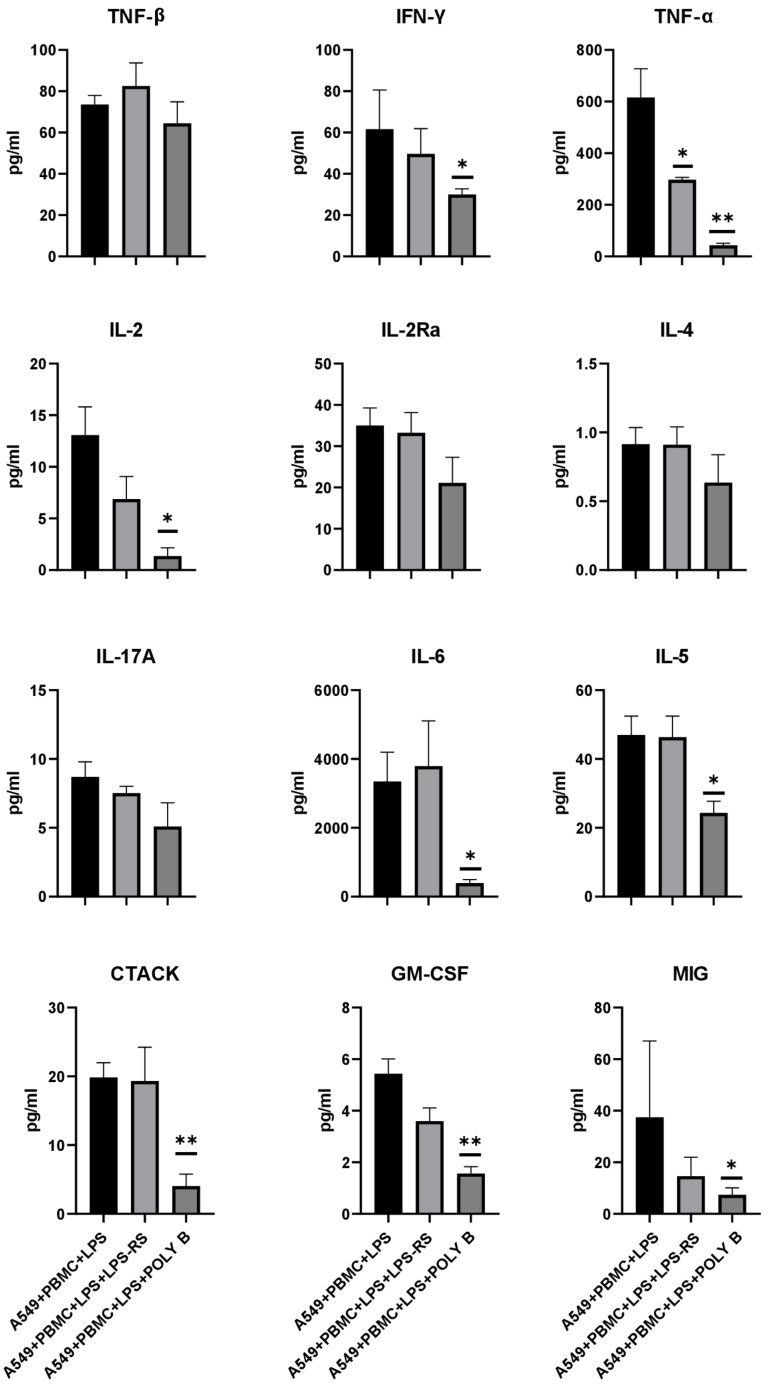
Influence of LPS-RS and polymyxin B on cytokine/chemokine concentrations in conditioned medium from the model system after 24 h treatment. Data are presented as a mean  ±  SEM, *n* = 3. * *p* < 0.05, ** *p* < 0.01; significance from the A549 + PBMC + LPS is assessed by one-way ANOVA with Tukey’s multiple comparisons test.

A Bio-plex analysis of cytokines and chemokines in a conditioned medium in the presence of polymyxin B from 48 analytes revealed a statistically significant change in eight analytes. Figure 8 shows the same analytes as for dexamethasone, while the remaining analytes are presented in Figure S4. Polymyxin B inhibited the production of TNF-α and IL-2 when A549 cells were cocultivated with leukocytes and LPS more than 10 times in comparison with model conditions. We also detected a reduced number of specific proinflammatory cytokines related to tissue-specific memory T-cells, such as CTACK being reduced more than twice times and macrophages GM-CSF and MIP-1a also by two times. On the other hand, it was surprising that the concentration of MIF responsible for M1 polarization of macrophages was significantly increased by almost 2 times under influence of polymyxin B (Figure S4). However, for LPS-RS, not so pronounced changes in the cytokine profile were detected. A significant decrease was found only for TNF-α. These data indicate that monitoring cell proliferation in real-time and multiplex cytokine analysis can be used either as a quantitative or comparative tool to assess the ability of drugs to reduce the production of cytokines and chemokines.

### 3.5. Influence of MSC and EVs on Cytokine/Chemokine Concentrations and Proliferation of A549 in Model System

**Figure 9:**
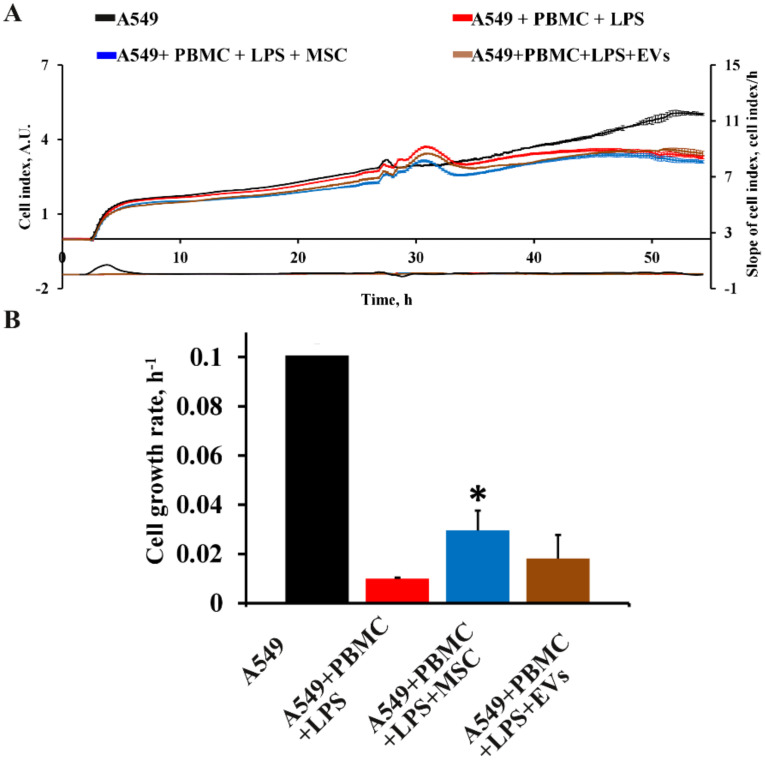
The effect of MSC and EVs on proliferation of A549 cells for 24 h in a model system. (**A**) The real-time curve of cell index for A549 cells and (**B**) the cell growth rate measured using RTCA iCELLigence for 35–50 h. The data are presented as a mean  ±  SEM, *n* = 3. * *p* < 0.05 vs. A549 + PBMC + LPS as determined by a one-way ANOVA with Tukey’s multiple comparison test.

**Figure 10:**
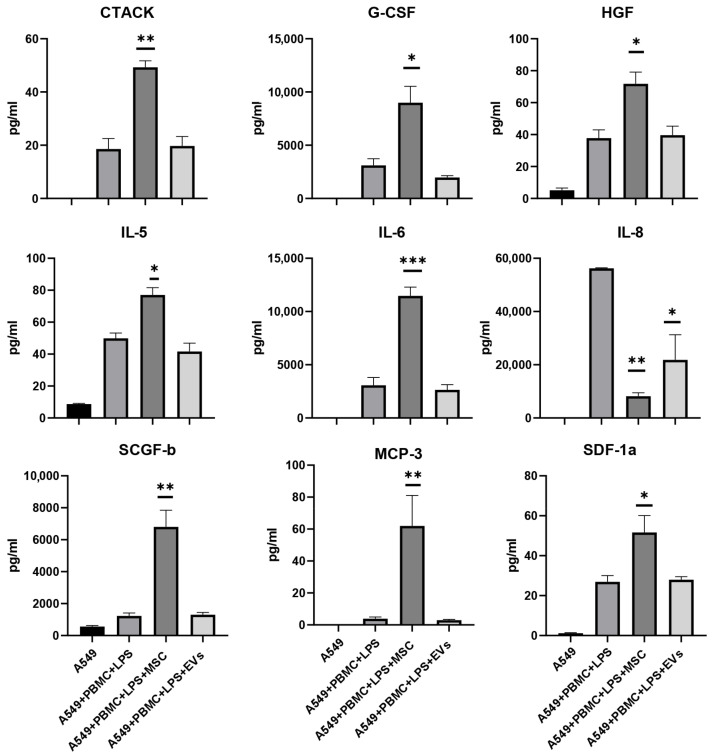
The effect of MSC and EVs on cytokine/chemokine concentrations in the conditioned medium from the model system after 24 h treatment. The data are presented as a mean  ±  SEM, n = 4. * *p* < 0.05, ** *p* < 0.01, *** *p* < 0.001 significance from A549 + PBMC + LPS is assessed by a one-way ANOVA with Tukey’s multiple comparison test.

In addition to pharmacological drugs, we tested MSC and EVs as effectors of the functional state of A549 cells and explored their ability to modulate the levels of cytokines/chemokines in the model system. MSC and EVs have been used due to their immunomodulatory, anti-inflammatory, and regenerative properties proven in the treatment of COVID-19 [34]. Analysis of cell growth curves for 24 h revealed the effect of MSC on an increase in cell index and cell growth rate by 30% compared to the A549+PBMC+LPS group (Figure 9A,B). EVs did not significantly affect the proliferation of A549, as well as the cytokine profile (Figure 9A,B and Figure 10).

To unravel the protective mechanisms of MSC and EVs, we conducted a Bio-plex analysis of the conditioned medium. The results of the analysis of the cytokine profile in the group of cocultivation with MSC showed the impossibility of unambiguous interpretation of the data as a shift towards the anti-inflammatory state. Conditioned medium contained both pro-inflammatory and anti-inflammatory cytokine/chemokines, e.g., the levels of CTACK, IL-5, and IL-6 were increased by more than two times while the levels of anti-inflammatory cytokine IL-8 decreased by more than two times in the presence of either MSC or EVs (Figure 10). Interestingly, only after cocultivation with MSC we found an increase in growth factors such as HGF (1.8 times), G-CSF (2.5 times), and SCGF-b (3 times), as well as chemokines MCP-3 (16 times) and SDF-1α (2 times), as compared with the A549 + PBMC + LPS group (Figure 10). The concentration of other analytes has not changed significantly (Figure S5). Thus, the results do not allow us to unambiguously interpret therapeutic effects of MSC in the model system.

## 4. Discussion

In this work, we have developed an in vitro model of pulmonary epithelial damage as a result of the induction of a cytokine storm associated with COVID-19 to unravel the molecular–cellular mechanisms of pulmonary epithelial damage and to test new therapeutic approaches for the treatment of complications of coronavirus infection. It is known that alveolar type II (ATII) cells play a key role as part of the distal lung epithelium, including roles in the innate immune response and are the main target for coronavirus SARS-CoV-2 infection [35,36]. These cells infection and the resulting damage leads to disruption of the alveolar-capillary barrier, lung edema, inflammation, ineffective gas exchange, impaired lung mechanics and reduced oxygenation, which resembles acute respiratory distress syndrome (ARDS) of other etiology [37]. In our model system, as lung epithelial cells, we used the human lung sarcoma cell line A549, which has the closest morpho-functional characteristics with ATII [38,39]. It should also be noted that PBMCs can be infected with SARS-CoV-2 and subsequently trigger cell-intrinsic innate immune responses [40,41]. Several studies have reported that hyper-inflammation accompanied by an increase in serum levels of pro-inflammatory cytokines and chemokines, leads to injury to alveolar epithelial cells and vascular endothelial cells [42], and also correlates with the severity of COVID-19 [43]. A significant contribution to the development of an uncontrolled hyper-inflammatory immune response and the secretion of proinflammatory cytokines can be made by leukocytes of human peripheral blood [44,45,46]. To simulate the proinflammatory microenvironment of pulmonary epithelial cells, we used PBMC activated by LPS. LPS is an agonist of TLR4, which triggers the expression of cytokines and chemokines in human PBMC through activation of the nuclear factor kappa B (NF-κB) signaling pathway (NF-κB) [47].

The concept of a cytokine storm is well-known and clinically proven. It is important to note that various entities can cause a cytokine storm, including not only infectious agents of viral or bacterial nature, but also CAR T-cell therapy and monogenic and autoimmune diseases [48]. A complex, interconnected network of cell types, signaling pathways, and cytokines is involved in cytokine storm disorders. IFN-γ, IL-1, IL-6, TNF-α, and IL-18 are key cytokines that often have elevated levels in cytokine storm and are believed to play a central immunopathologic roles. Although viruses are considered the most common infectious triggers of the cytokine storm, a number of bacterial infections or pathogen-associated molecular patterns (PAMPs) can trigger an uncontrolled immune response [49]. The immune response to various coronaviruses or bacteria can lead to the appearance of different cytokine profiles, however, clinical symptoms and immune responses in patients are similar [50]. Thus, the induction of a hyperinflammatory response can have a different nature, whereas the effector part of the reaction can be carried out using through common molecular and cellular mechanisms. Despite the possibility of the induction of a cytokine storm by various activators of innate immunity, the use of SARS-CoV-2 is undoubtedly more relevant for accurate reproduction of pathological cascades. However, working with the live SARS-CoV-2 virus imposes significant limitations associated with increased requirements for laboratory equipment in terms of biosafety, which greatly increases the cost, complicates research, and slows down progress in finding therapeutic approaches to treat the effects of COVID-19. The use of PAMPs may be one of the approaches to trigger an inflammatory response in immune cells. Indeed, an increase in cytokines such as TNF-α, MCP-1, IL-6, IL-1β, and IL-10 was shown in the rat ARDS model induced by LPS administration, which corresponds to the cytokine profile of patients with a severe form of COVID-19 [51]. Moreover, the introduction of LPS also activates similar signaling pathways, in particular the AT1R/JAK/STAT signaling pathway and the assembly of NLRP3 in the inflammasome in infected/damaged lung tissue cells and resident macrophages, which mimics moderate and severe COVID-19 pathology [52,53]. Thus, the induction of a hyperinflammatory LPS response can be used to model the pathogenetic mechanisms of ARDS observed in COVID-19 [54,55]. The limitation of our study is the use of PBMCs from healthy donors, as well as the use of LPS instead of SARS-CoV-2 particles or its components. However, the approach proposed allows for a broader set of research manipulations, which are limited in the case of the content of an infectious agent in the model system due to biological safety considerations.

For our purpose, it was essential to create conditions similar to a cytokine storm. A key parameter for the success of modeling a hyper-inflammatory reaction in vitro should be an increase in the concentration of cytokines and chemokines in a conditioned culture medium regardless of the way leukocytes are activated. We used the MILLIPLEX^®^ human cytokine/Chemokine/Growth kit for 48 individual immune factors that allowed us to evaluate pro-inflammatory factors in our model system. A significant increase in 40 analytes was identified, including IL-1, IL-2, IL-6, IL-10, IFN-γ, and TNF-α, which are important proinflammatory cytokines that have been established in patients with COVID-19 [56,57]. In addition to chemokines, RANTES, GRO-α, and MIP-1b were found to be associated with the severity of COVID-19 [58,59,60]. Based on the cytokine profile data, we can assume that in the developed model, the inflammatory response affects two main branches of the immune response: innate, represented mostly by neutrophils and, to a lesser extent, macrophages, as well as the cellular response implemented through T-lymphocytes. Thus, the profile of cytokines and chemokines observed in the developed model corresponds to a similar profile in the blood of patients with severe COVID-19 [30,61].

We assumed that it is the excessive production of cytokines that is responsible for damage to the lung epithelium in COVID-19-mediated ARDS; therefore, we assessed the impact of the pro-inflammatory microenvironment on A549 cells in our model using the iCELLigence real-time cell analysis system [62]. This system can be used for the label-free real-time monitoring of A549 cell proliferation, viability and cytotoxicity, that is, the main parameters that are subject to change during cytotoxic challenge. Moreover, if an infectious agent is used instead of LPS, this approach makes it possible to remotely analyze cell growth without the participation of an operator, which reduces biological risks. Indeed, co-cultivation of A549 cells with activated PBMC for 24 h led to a sharp decrease in the cellular index, which was associated with both the cellular death of pulmonary epithelial cells and a decrease in their proliferation rate. It should be noted that the treatment of A549 cells with LPS only did not cause significant changes, which indicates the dominant role of factors produced by leukocytes in damage to the lung epithelium. Thus, in our model, the assessment of the cellular index and its derivatives was a reliable tool for assessing the damaging effect of hyperinflammation, which can be used to analyze therapeutic agents.

It should be noted that the level of cell death in the developed model system was only about 10% of cells from the entire A549 population. It has been shown that cell death of ATII cells in patients with COVID-19 occurs by PANoptosis. TNF-α and IFN-γ co-treatment activated the JAK/STAT1/IRF1 axis, inducing nitric oxide production and driving caspase-8/FADD-mediated PANoptosis [63]. Since we also observed a significant increase in TNF-α and IFN-γ in the conditioned medium of the developed model, we assumed that they could induce PANoptosis in A549 cells. To test this hypothesis, we evaluated changes in PANoptosis markers in A549 cells. An increase in the content of a few markers of apoptosis, caspase-3 and -8, and Bax; components of the inflammasome RIPK1 and RIPK3; as well as a marker of necroptosis—pMLKL—was revealed, which may indicate the launch of PANoptotic death of A549 cells [64,65].

One of the causes of necrotic or regulated cell death (apoptotic, autophagic) may be oxidative stress [66], which is also observed during inflammation. Cytokine storm triggers the extra production of ROS in cells of the immune system to destroy the antigen, but these species adversely oxidize cellular lipids, proteins, and DNA and ultimately ignite the programmed death of lung epithelial cells and endothelium [67,68]. A recent study of patients with COVID-19 showed elevated levels of lipid peroxidation products such as malondialdehyde (MDA) in their plasma, which was associated with oxidative stress and may be an important diagnostic signature of a severe form of the disease [69]. The analysis of oxidative stress in A549 cells after 24 h of co-cultivation with activated PBMC revealed both oxidative damage to cytoplasmic membranes and ROS production in mitochondria. Thus, our model system reproduces the pathological molecular and cellular mechanisms of damage to the pulmonary epithelium observed in patients with COVID-19, and oxidative stress parameters can also be used to assess the mechanisms of lung epithelial death and therapeutic approaches.

A model reproducing elements of the pathological cascade of cytotoxic damage to the pulmonary epithelium should respond to therapeutic effects with drugs having molecular targets in this pathological pathway. We selected five therapeutic approaches, three of which were represented by pharmacological agents with anti-inflammatory properties (dexamethasone, LPS-RS, and polymyxin B), while two other therapeutic options were based on the use of cellular technologies. We chose well-studied agents in order to demonstrate the possibility of evaluating their therapeutic effectiveness in a model system. An increase in the cellular index and a decrease in pro-inflammatory cytokines in the conditioned environment can supplement the identified anti-inflammatory effect for dexamethasone and polymyxin B. In general, for these drugs, the results of the studies are consistent with previously described models simulating pyelonephritis [70].

Evaluation of the anti-inflammatory effects of agents was executed through their direct addition to the chamber where A549 cells were co-cultured with activated PBMC. However, to study the effects of MSC, we had to modify the test system and introduced the cells by inserts with 0.4 µm pore polyester membrane, since the direct addition of MSC to the test system would affect the cellular impedance and would make it impossible to assess the proliferation of A549 cells. It has been assumed that MSCs implement their therapeutic mechanisms to a greater extent through the secretion of extracellular vesicles [71], which could enter the test system by travelling through the pores of polyester membrane. MSCs are known to acquire reparative and immunoregulatory properties. Moreover, numerous studies on animal models show their high effectiveness in reducing the inflammatory response in acute pulmonary injury [72,73]. However, our data demonstrate an increase in pro-inflammatory cytokines/chemokines with a simultaneous increase in the concentration of growth factors in a conditioned medium. In addition, the increase in the cell index was not so significant compared to dexamethasone and polymyxin B. Thus, we could not unambiguously interpret the results obtained in our model system and draw conclusions about the anti-inflammatory effect of MSCs. MSCs are a complex living system with a pleiotropic therapeutic effect that can only be fully accomplished and analyzed in model systems in vivo. For example, we found a significant statistically significant increase in the level of growth factors (TGF, G-CSF) observed only in the group treated with MSC. In addition, HGF promotes active regeneration of lung tissue and prevents its fibrosis [74]; in turn, G-CSF also prevents lung fibrosis by inducing directed migration of bone marrow cells into the damaged area of lung tissue [75]. The data obtained clearly demonstrate the dual nature of the action of MSC in the inflammatory environment. On the one hand, it prevents an uncontrolled immune response from developing, and on the other, it limits the complete inhibition of the immune response. In addition, MSC increased the level of SGF-β, which promotes the proliferation and differentiation of hematopoietic stem cells thus indicating a possible protective role of MSC through preservation the pool of immune cells from depletion, which is observed in severe and moderate forms of COVID-19.

An interesting fact was the absence of any significant effects of EVs in our test system, despite the fact that MSCs were cultured through porous membranes and their effect can only be explained by the secretion of humoral factors, including EVs. It is known that the contents of EVs depend on the microenvironment of MSC, and accordingly, in the inflammatory microenvironment, MSCs can change the spectrum of secreted factors [76]. MSCs were also affected by LPS, which was present in a culture medium and could additionally modulate the functions of the MSCs. It is known that the survival of MSCs treated with LPS is elevated due to the induction of anti-apoptotic mechanisms within [77,78]. While EVs were isolated from the conditioned medium under standard conditions of cultivation of MSCs, it can be assumed that EVs cannot be carriers of all the therapeutic properties of MSCs, displaying a high level of plasticity of their phenotype and that they are always “adjusting” themselves to the microenvironment.

## 5. Conclusions

We developed a specific in vitro test system based on human cells which allows for evaluating the effectiveness of therapeutic approaches for correcting excessive inflammatory response (cytokine storm) and can be used for screening potential therapeutic drugs against complications of COVID-19. The results of the study demonstrated that in the case of biotechnological therapeutic approaches (such as stem cells), the use of an in vitro test system may not be effective enough due to the multifactorial nature of the action exerted by stem cells, and additional studies in the in vivo system are needed.

## Data Availability

The data that support the findings of this study are available from the corresponding author upon reasonable request.

## Supplementary Materials

The following supporting information can be downloaded at: https://www.mdpi.com/article/10.3390/antiox11101910/s1, **Figure S1.** An effect on cell index of A549 cells co-culture with PBMC or treatment with LPS (5 µg/mL). **Figure S2.** Spider plot, cytokine/chemokine concentrations (pg/mL; log scale) in conditioned medium 24 h after A549, PBMC, A549 cocultivation with PBMC or PBMC primed with LPS; n = 5, ** p* < 0.5, *** p* < 0.001, **** p* < 0.0001, **** *p* < 0.00001 significance from A549 + PBMC is assessed by a one-way ANOVA with Tukey’s multiple comparison test. **Figure S3.** Supplemental data for effect of dexamethasone on cytokine/chemokine concentrations in the conditioned medium from the model system after 24 h treatment. The data are presented as a mean  ±  SEM, n = 3. ** p* < 0.05, *** p* < 0.01, *** p* < 0.001, the significance from the A549+PBMC+LPS is assessed by a one-way ANOVA with the Tukey’s multiple comparisons test. **Figure S4.** Supplemental data on the effect of LPS-RS and polymyxin B on cytokine/chemokine concentrations in conditioned medium from the model system after 24 h treatment. Data are presented as a mean  ±  SEM, n = 3. *** p* < 0.01, **** p* < 0.001, significance from the A549+PBMC+LPS is assessed by one-way ANOVA with Tukey’s multiple comparisons test. **Figure S5.** Supplemental data for effect of MSC and EVs on cytokine/chemokine concentrations in the conditioned medium from the model system after 24 h treatment. The data are presented as a mean  ±  SEM, n = 4.
