# Supplementary material for: Development of an In Vitro Model of SARS-CoV-Induced Acute Lung Injury for Studying New Therapeutic Approaches
Source: Antioxidants (Basel). 2022 Sep 27;11(10):1910. doi: 10.3390/antiox11101910 (PMC9598130; doi:10.3390/antiox11101910)
Supplement: Supplementary file 1 [file antioxidants-11-01910-s001.zip › antioxidants-1908877-Figure S3.pdf]

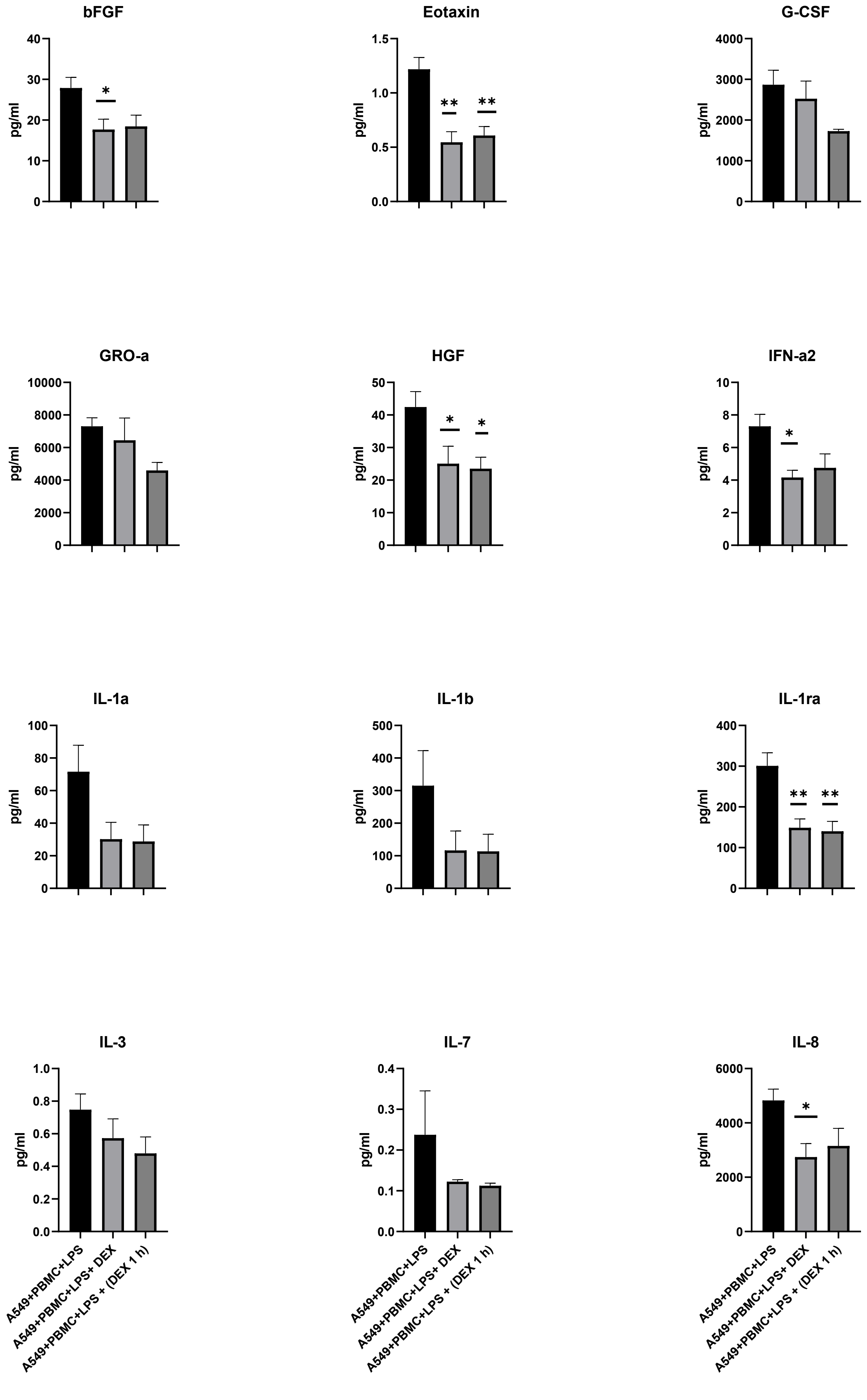

bFGF
Eotaxin
G-CSF
GRO-a
HGF
IFN-a2
IL-1a
IL-1b
IL-1ra
IL-3
IL-7
IL-8
pg/ml
*
**
A549+PBMC+LPS
A549+PBMC+LPS+ DEX
A549+PBMC+LPS + (DEX 1 h)

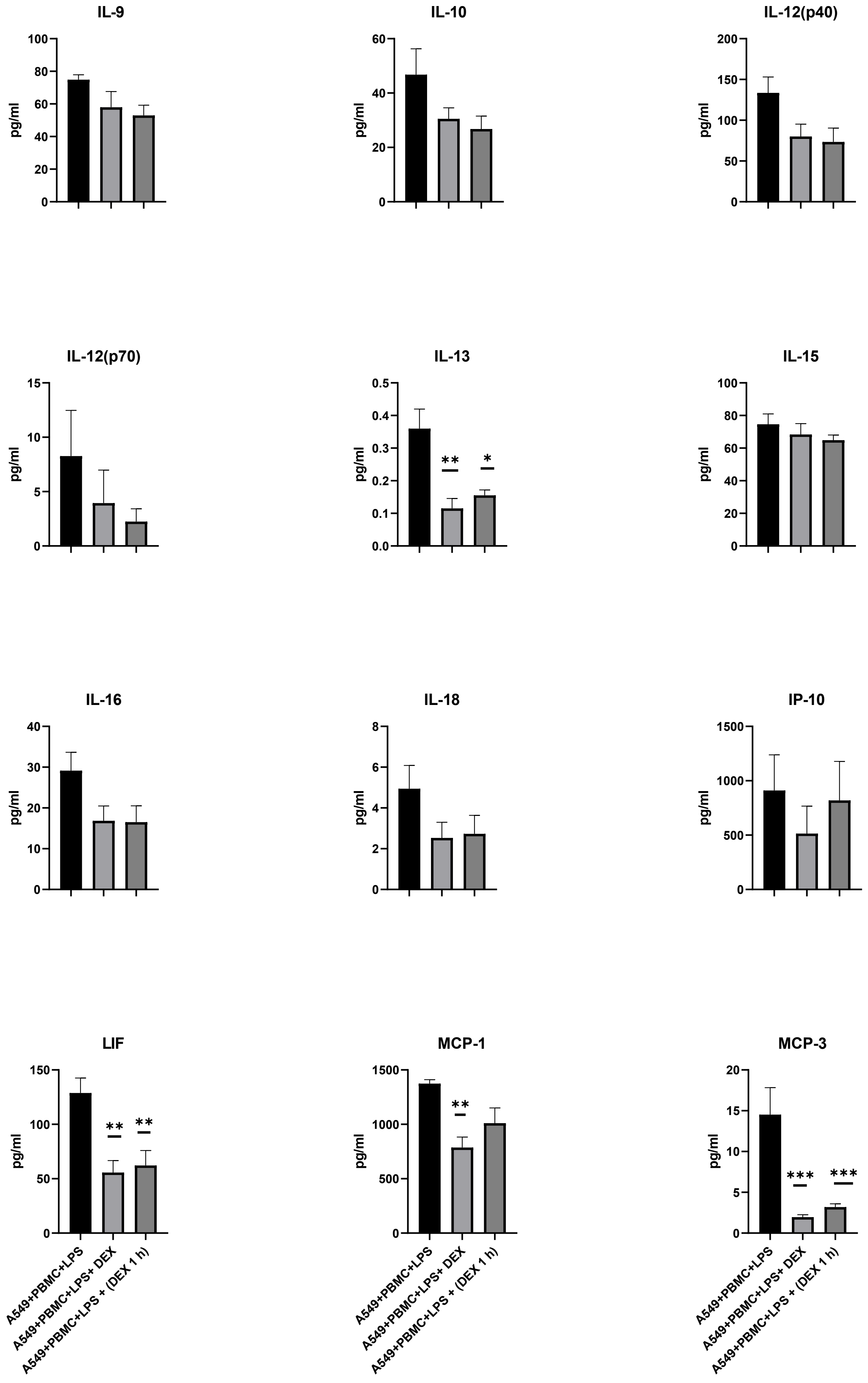
IL-9
pg/ml
0
20
40
60
80
100
IL-10
pg/ml
0
20
40
60
IL-12(p40)
pg/ml
0
50
100
150
200
IL-12(p70)
pg/ml
0
5
10
15
IL-13
pg/ml
0.0
0.1
0.2
0.3
0.4
0.5
**
*
IL-15
pg/ml
0
20
40
60
80
100
IL-16
pg/ml
0
10
20
30
40
IL-18
pg/ml
0
2
4
6
8
IP-10
pg/ml
0
500
1000
1500
LIF
pg/ml
0
50
100
150
**
**
MCP-1
pg/ml
0
500
1000
1500
**
MCP-3
pg/ml
0
5
10
15
20
***
***
A549+PBMC+LPS
A549+PBMC+LPS+ DEX
A549+PBMC+LPS + (DEX 1 h)

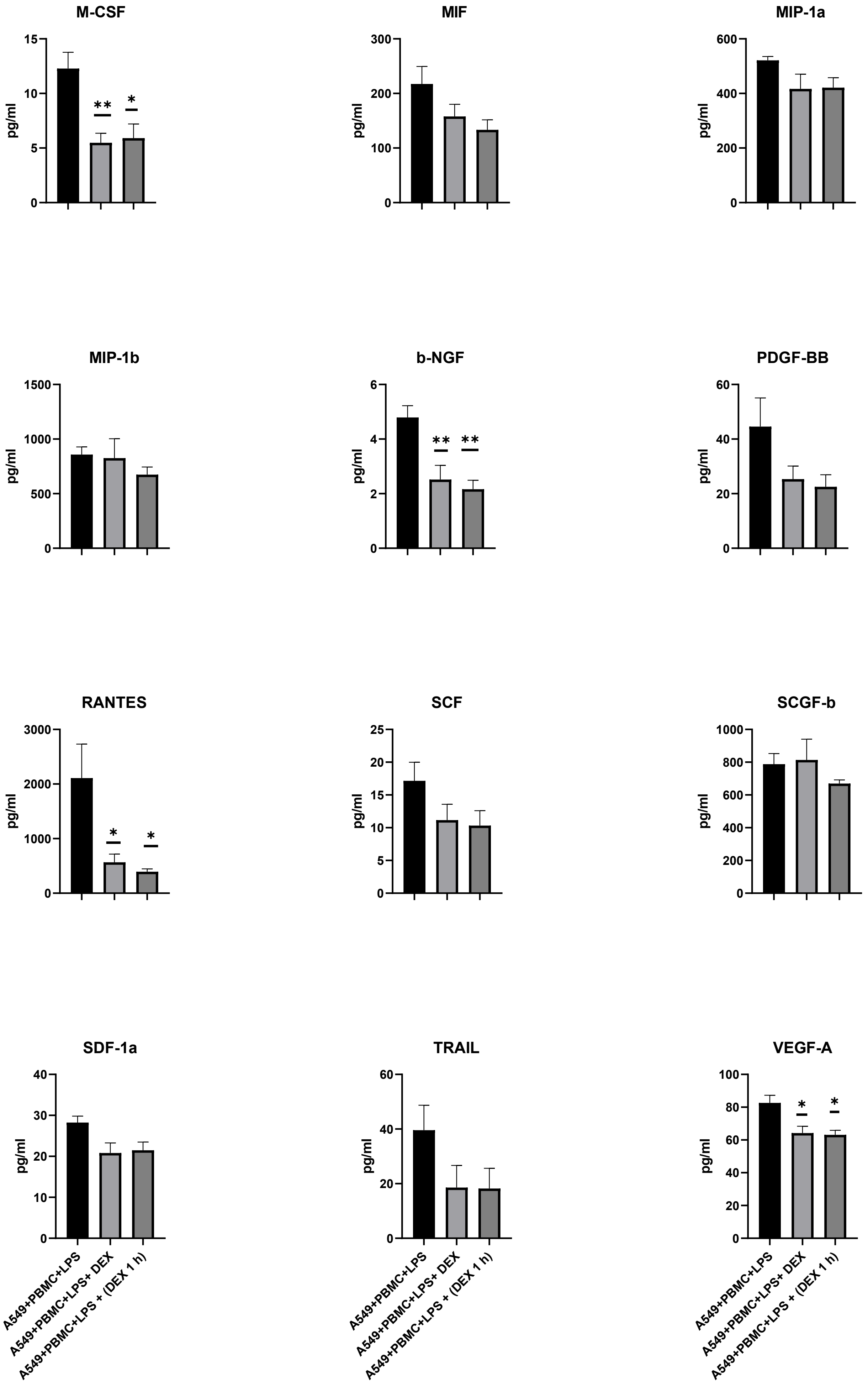
M-CSF
15
10
5
0
pg/ml
**
*
MIF
300
200
100
0
pg/ml
MIP-1a
600
400
200
0
pg/ml
MIP-1b
1500
1000
500
0
pg/ml
b-NGF
6
4
2
0
pg/ml
**
**
PDGF-BB
60
40
20
0
pg/ml
RANTES
3000
2000
1000
0
pg/ml
*
*
SCF
25
20
15
10
5
0
pg/ml
SCGF-b
1000
800
600
400
200
0
pg/ml
SDF-1a
40
30
20
10
0
pg/ml
TRAIL
60
40
20
0
pg/ml
VEGF-A
100
80
60
40
20
0
pg/ml
*
*
A549+PBMC+LPS
A549+PBMC+LPS+ DEX
A549+PBMC+LPS + (DEX 1 h)
