# Supplementary material for: Development of an In Vitro Model of SARS-CoV-Induced Acute Lung Injury for Studying New Therapeutic Approaches
Source: Antioxidants (Basel). 2022 Sep 27;11(10):1910. doi: 10.3390/antiox11101910 (PMC9598130; doi:10.3390/antiox11101910)
Supplement: Supplementary file 1 [file antioxidants-11-01910-s001.zip › antioxidants-1908877-Figure S4.pdf]

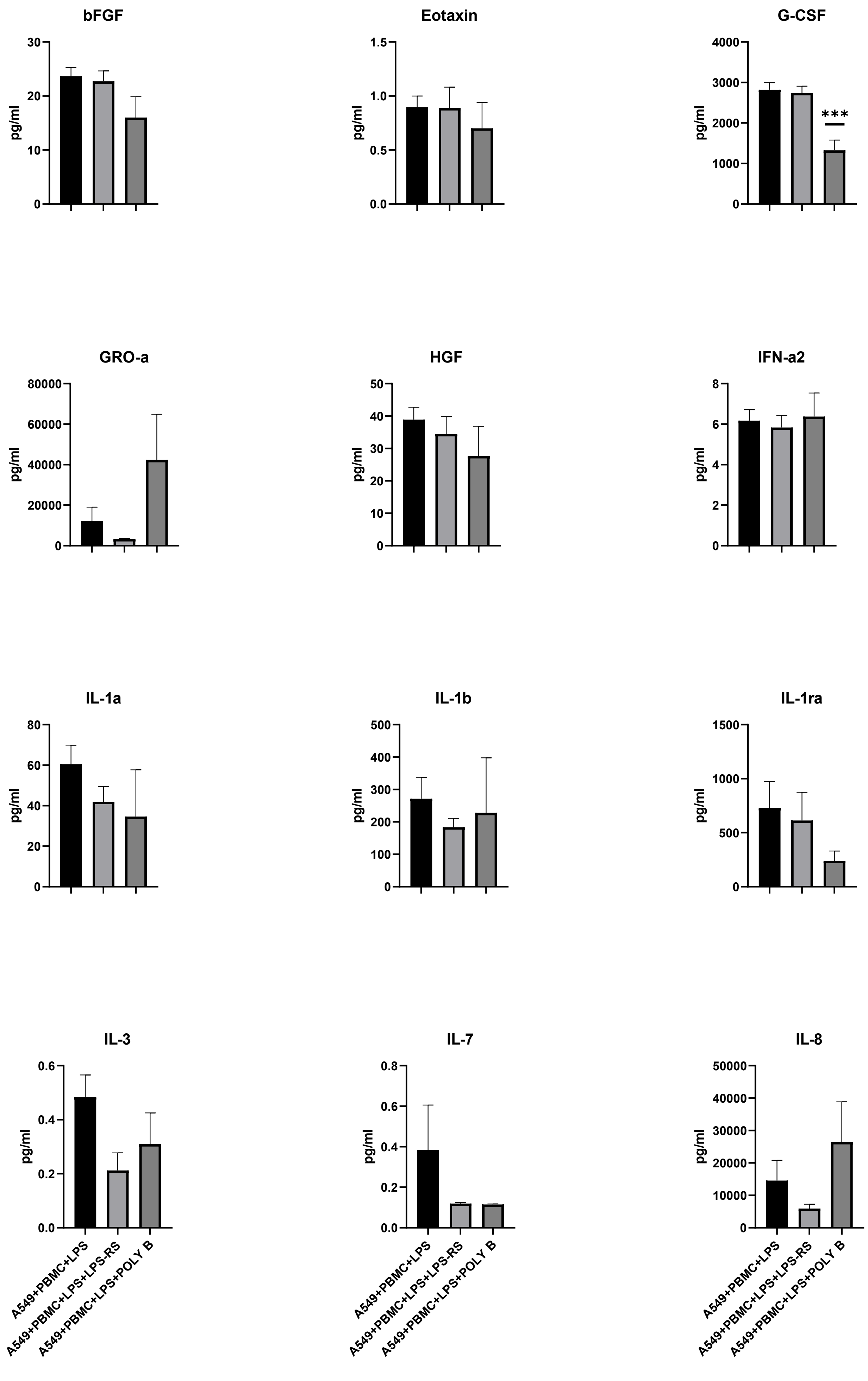

bFGF
Eotaxin
G-CSF
***
GRO-a
HGF
IFN-a2
IL-1a
IL-1b
IL-1ra
IL-3
IL-7
IL-8
pg/ml
A549+PBMC+LPS
A549+PBMC+LPS+LPS-RS
A549+PBMC+LPS+POLY B

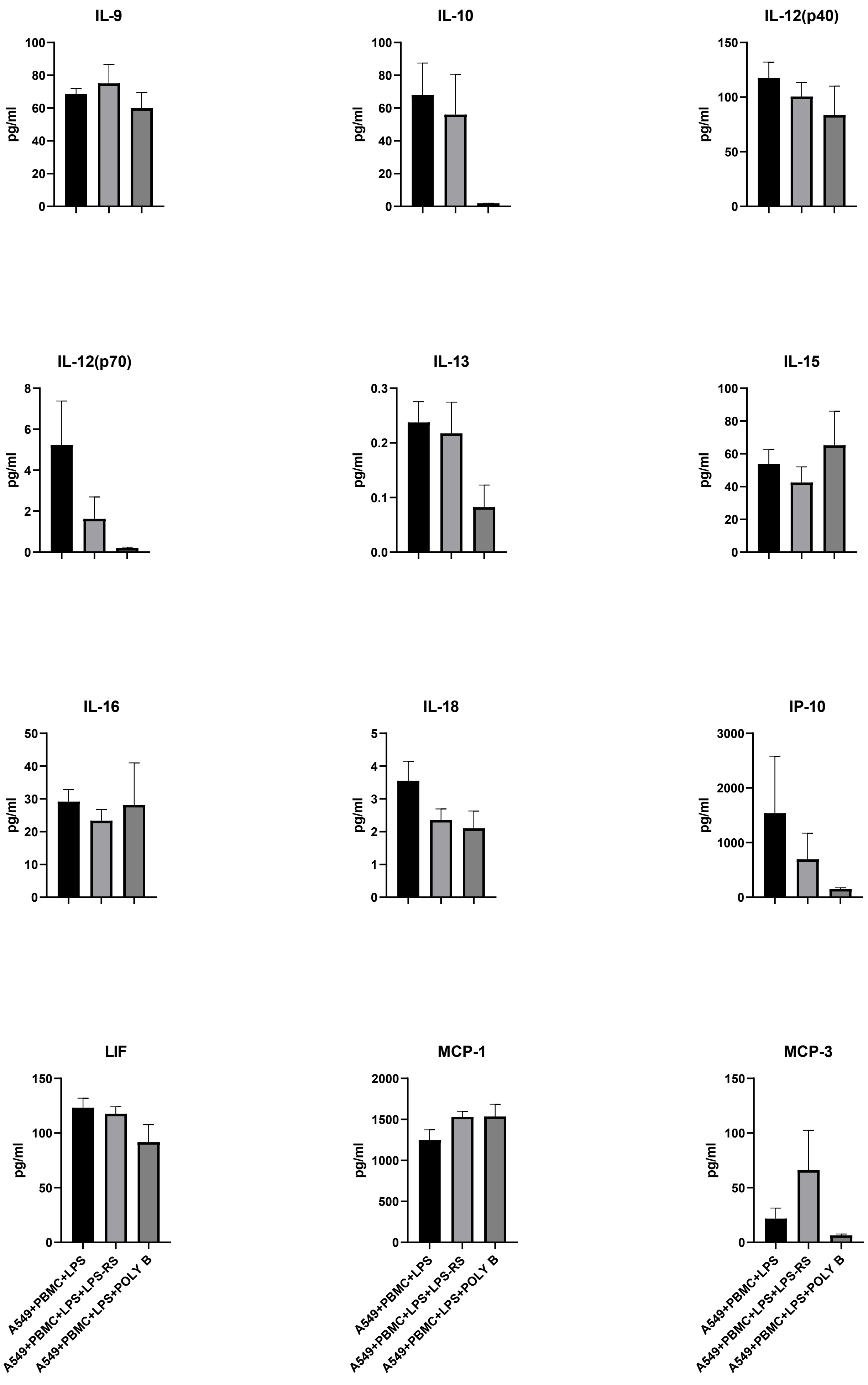

IL-9
IL-10
IL-12(p40)
IL-12(p70)
IL-13
IL-15
IL-16
IL-18
IP-10
LIF
MCP-1
MCP-3
pg/ml
0
20
40
60
80
100
50
150
2
4
6
8
0.0
0.1
0.2
0.3
10
30
1
3
5
1000
2000
3000
500
1500
A549+PBMC+LPS
A549+PBMC+LPS+LPS-RS
A549+PBMC+LPS+POLY B

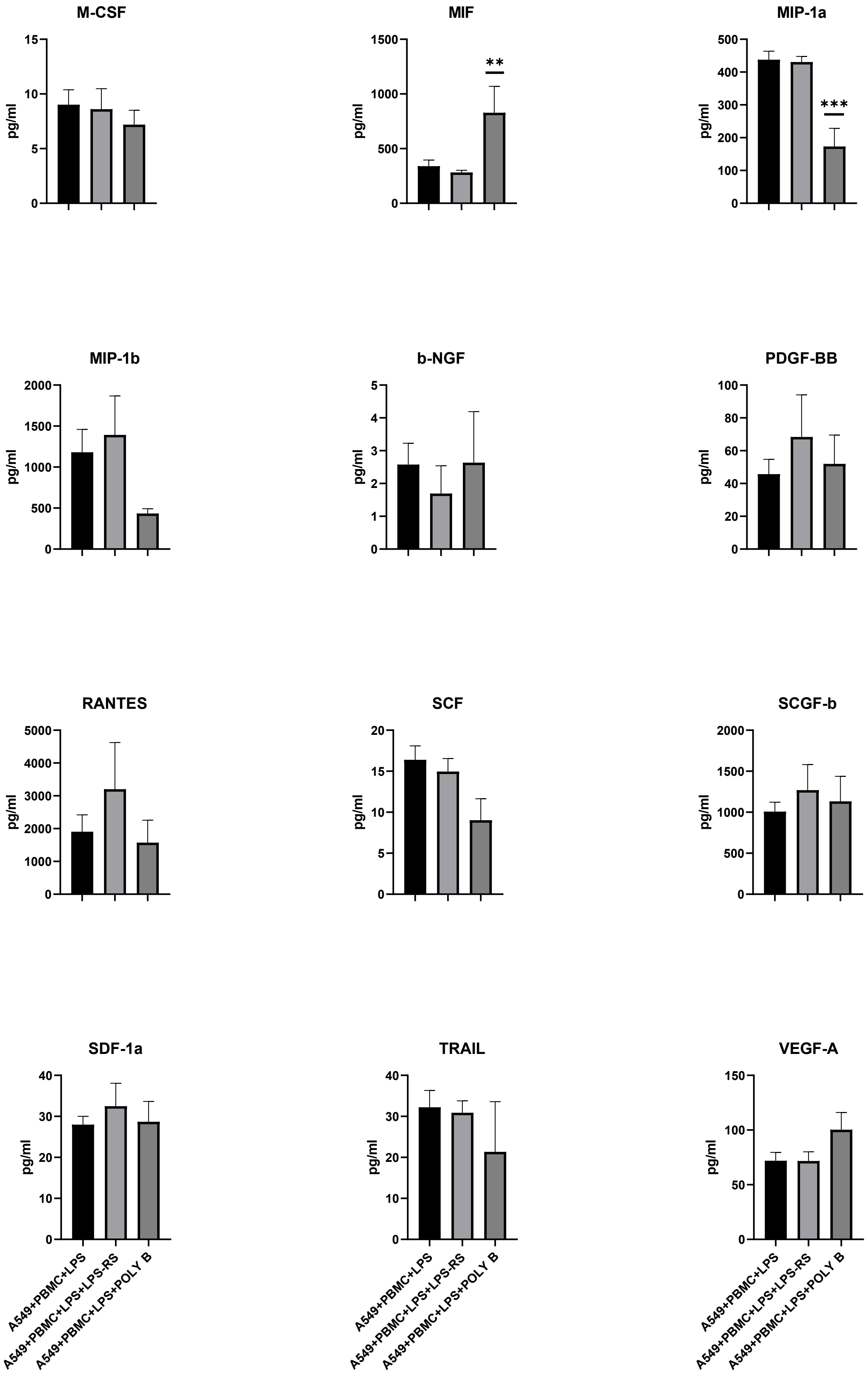

M-CSF
MIF
**
MIP-1a
***
MIP-1b
b-NGF
PDGF-BB
RANTES
SCF
SCGF-b
SDF-1a
TRAIL
VEGF-A
pg/ml
A549+PBMC+LPS
A549+PBMC+LPS+LPS-RS
A549+PBMC+LPS+POLY B
