# Supplementary material for: Development of an In Vitro Model of SARS-CoV-Induced Acute Lung Injury for Studying New Therapeutic Approaches
Source: Antioxidants (Basel). 2022 Sep 27;11(10):1910. doi: 10.3390/antiox11101910 (PMC9598130; doi:10.3390/antiox11101910)
Supplement: Supplementary file 1 [file antioxidants-11-01910-s001.zip › antioxidants-1908877-Figure S5.pdf]

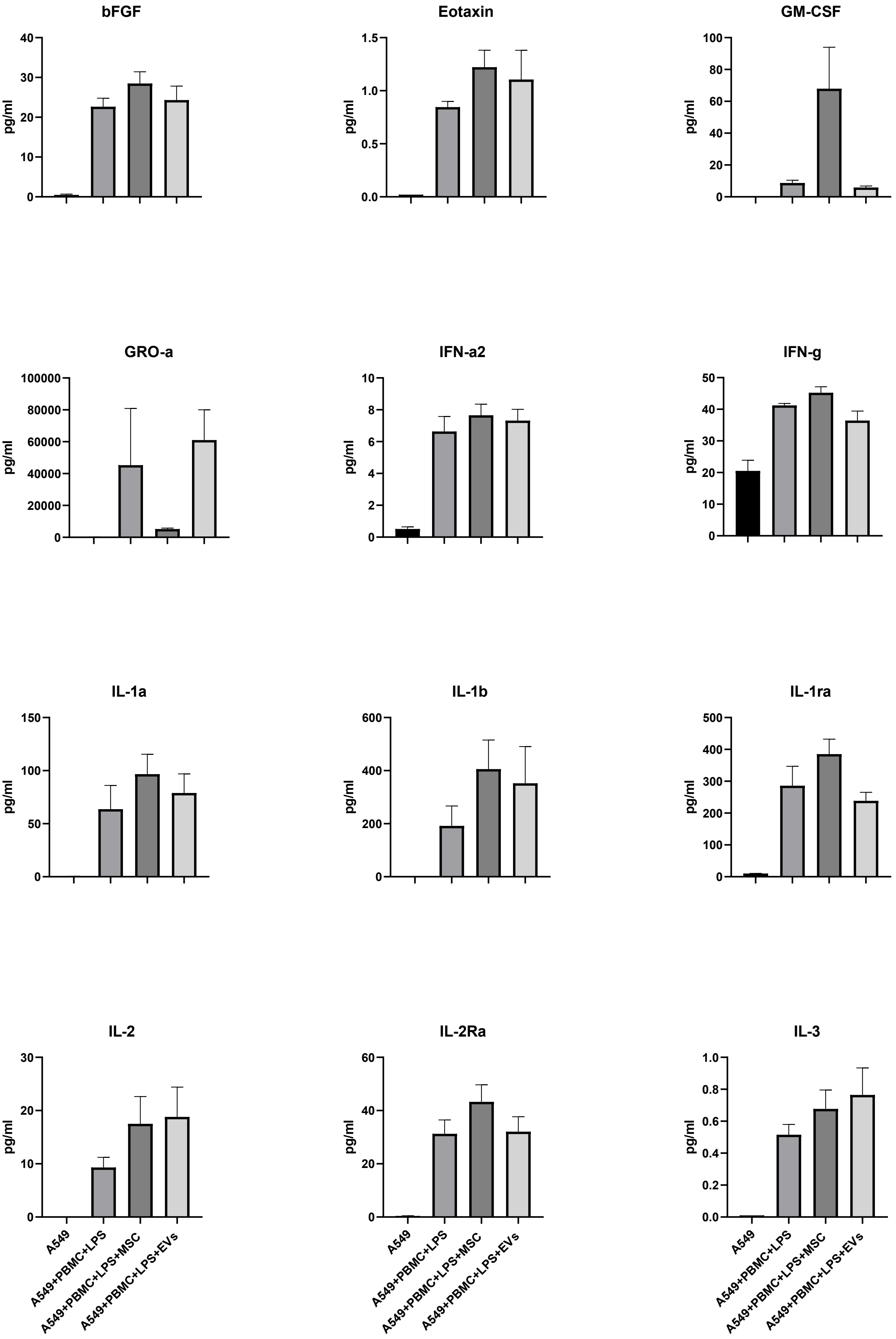

bFGF
Eotaxin
GM-CSF
GRO-a
IFN-a2
IFN-g
IL-1a
IL-1b
IL-1ra
IL-2
IL-2Ra
IL-3
pg/ml
A549
A549+PBMC+LPS
A549+PBMC+LPS+MSC
A549+PBMC+LPS+EVs

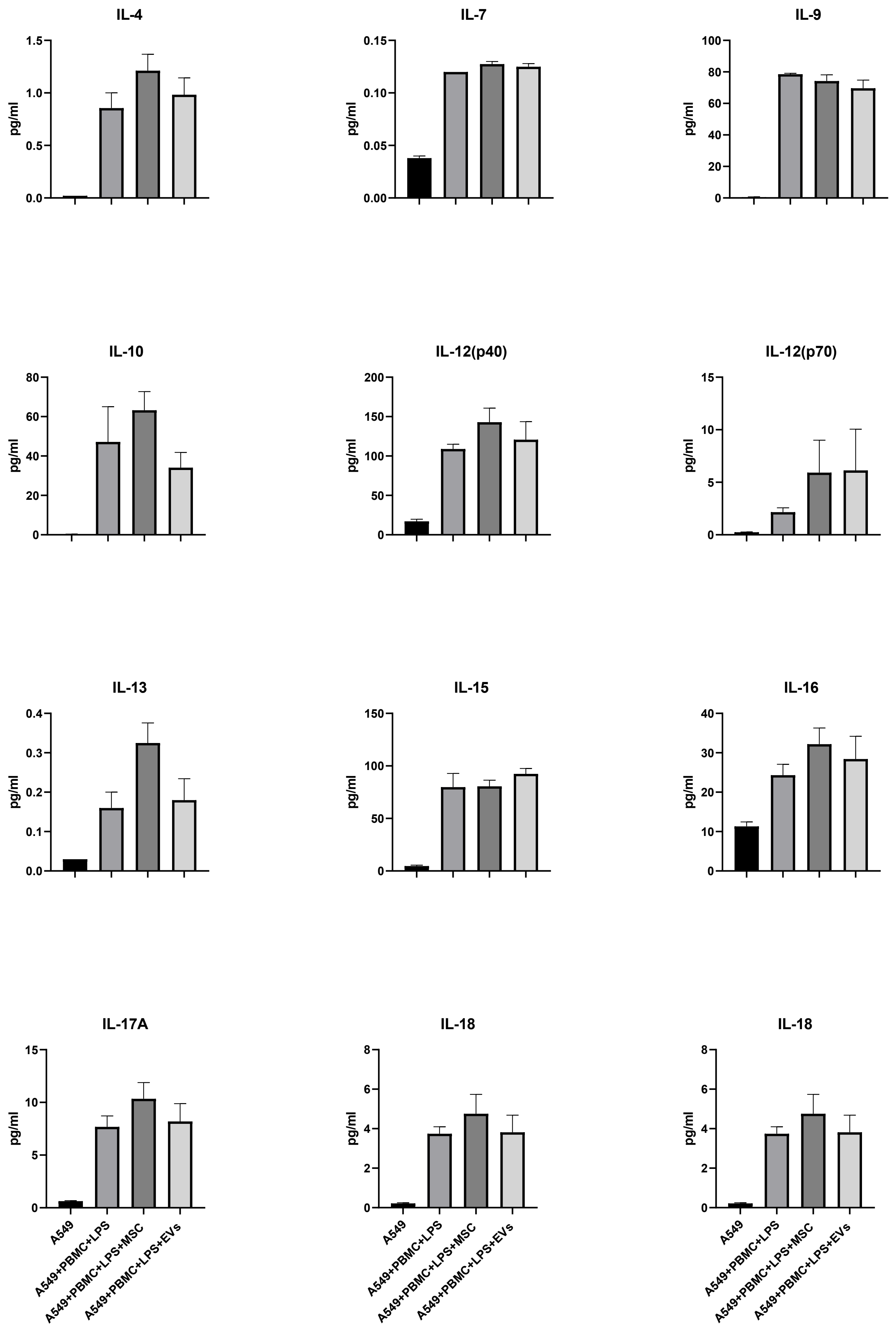

IL-4
IL-7
IL-9
IL-10
IL-12(p40)
IL-12(p70)
IL-13
IL-15
IL-16
IL-17A
IL-18
IL-18
pg/ml
A549
A549+PBMC+LPS
A549+PBMC+LPS+MSC
A549+PBMC+LPS+EVs

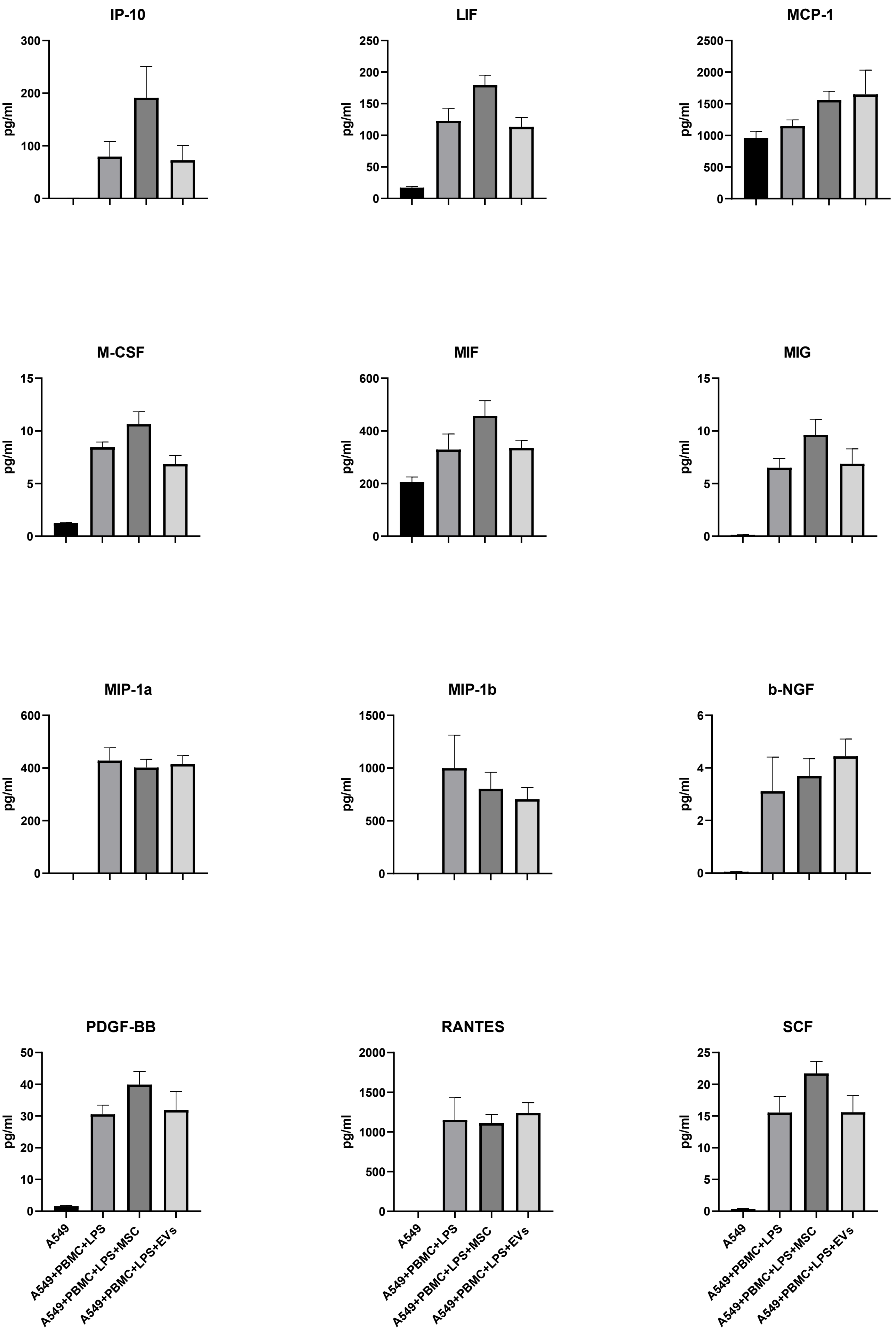
IP-10
pg/ml
0
100
200
300
LIF
pg/ml
0
50
100
150
200
250
MCP-1
pg/ml
0
500
1000
1500
2000
2500
M-CSF
pg/ml
0
5
10
15
MIF
pg/ml
0
200
400
600
MIG
pg/ml
0
5
10
15
MIP-1a
pg/ml
0
200
400
600
MIP-1b
pg/ml
0
500
1000
1500
b-NGF
pg/ml
0
2
4
6
PDGF-BB
pg/ml
0
10
20
30
40
50
RANTES
pg/ml
0
500
1000
1500
2000
SCF
pg/ml
0
5
10
15
20
25
A549
A549+PBMC+LPS
A549+PBMC+LPS+MSC
A549+PBMC+LPS+EVs

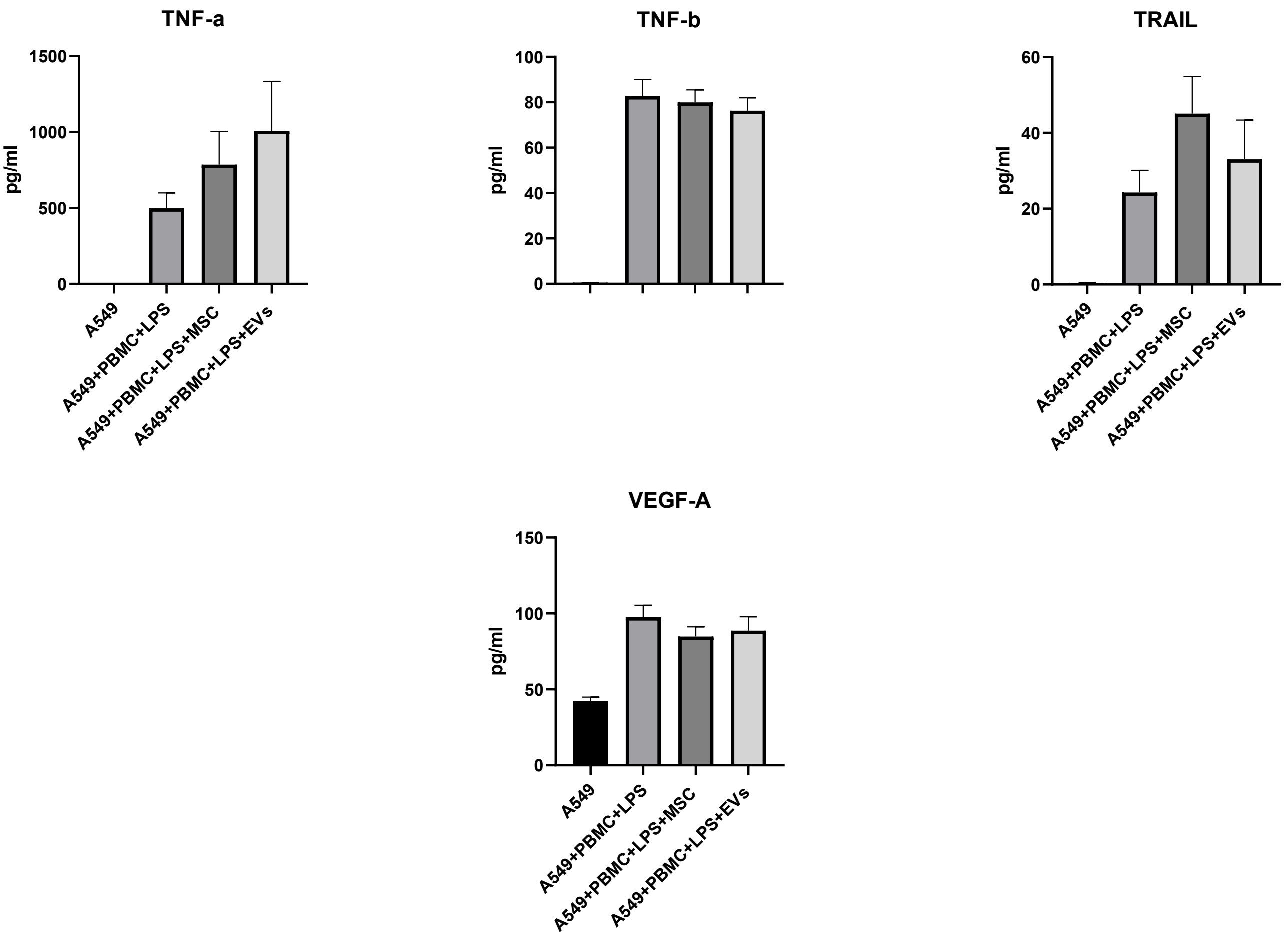

TNF-a
pg/ml
1500
1000
500
0
A549
A549+PBMC+LPS
A549+PBMC+LPS+MSC
A549+PBMC+LPS+EVs
TNF-b
pg/ml
100
80
60
40
20
0
TRAIL
pg/ml
60
40
20
0
A549
A549+PBMC+LPS
A549+PBMC+LPS+MSC
A549+PBMC+LPS+EVs
VEGF-A
pg/ml
150
100
50
0
A549
A549+PBMC+LPS
A549+PBMC+LPS+MSC
A549+PBMC+LPS+EVs
