# Supplementary figures and images for: Development of an In Vitro Model of SARS-CoV-Induced Acute Lung Injury for Studying New Therapeutic Approaches
Source: Antioxidants (Basel). 2022 Sep 27;11(10):1910. doi: 10.3390/antiox11101910 (PMC9598130; doi:10.3390/antiox11101910)

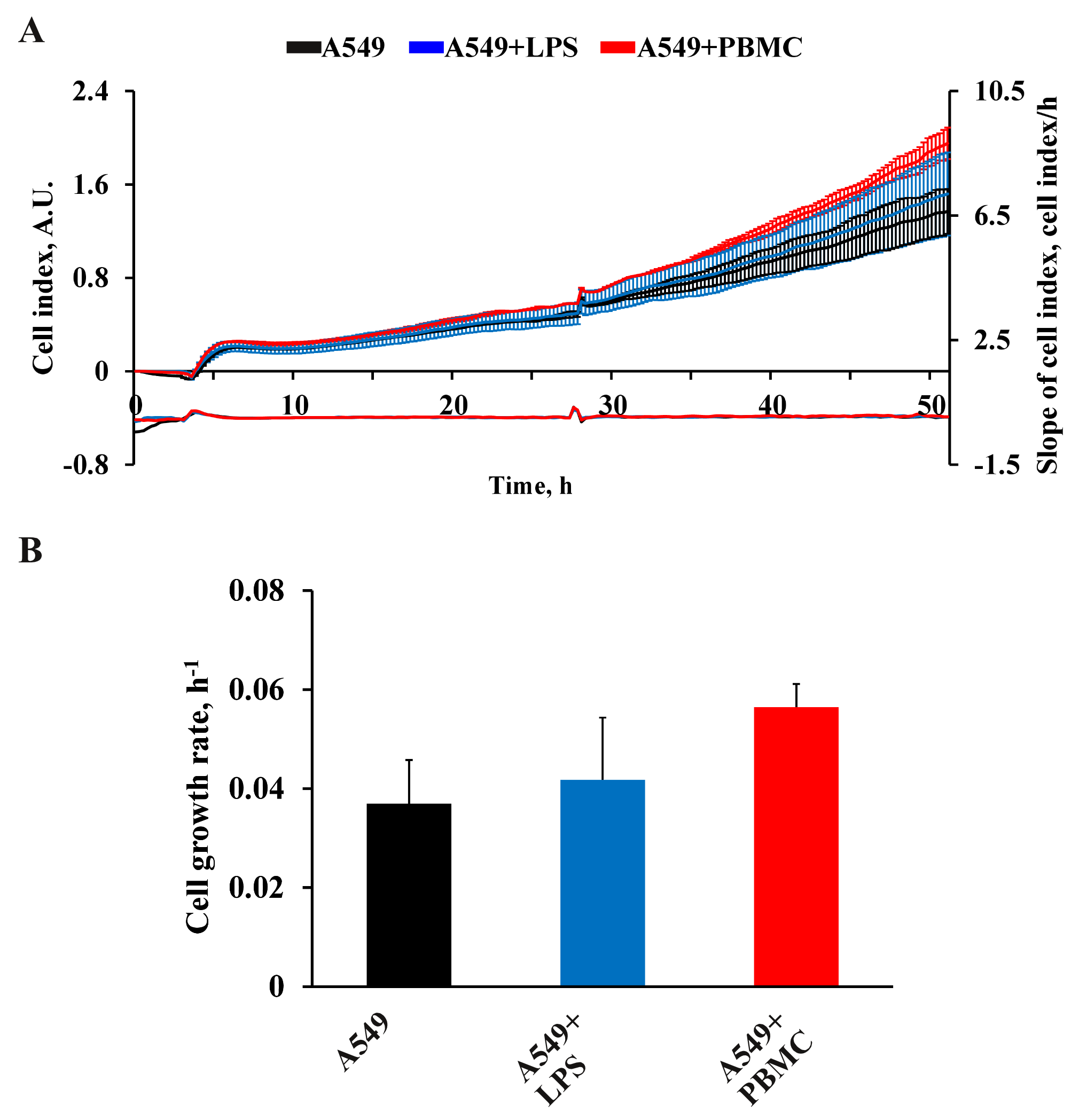

Supplement: Supplementary file 1 [file antioxidants-11-01910-s001.zip › antioxidants-1908877-Figure S1.png]

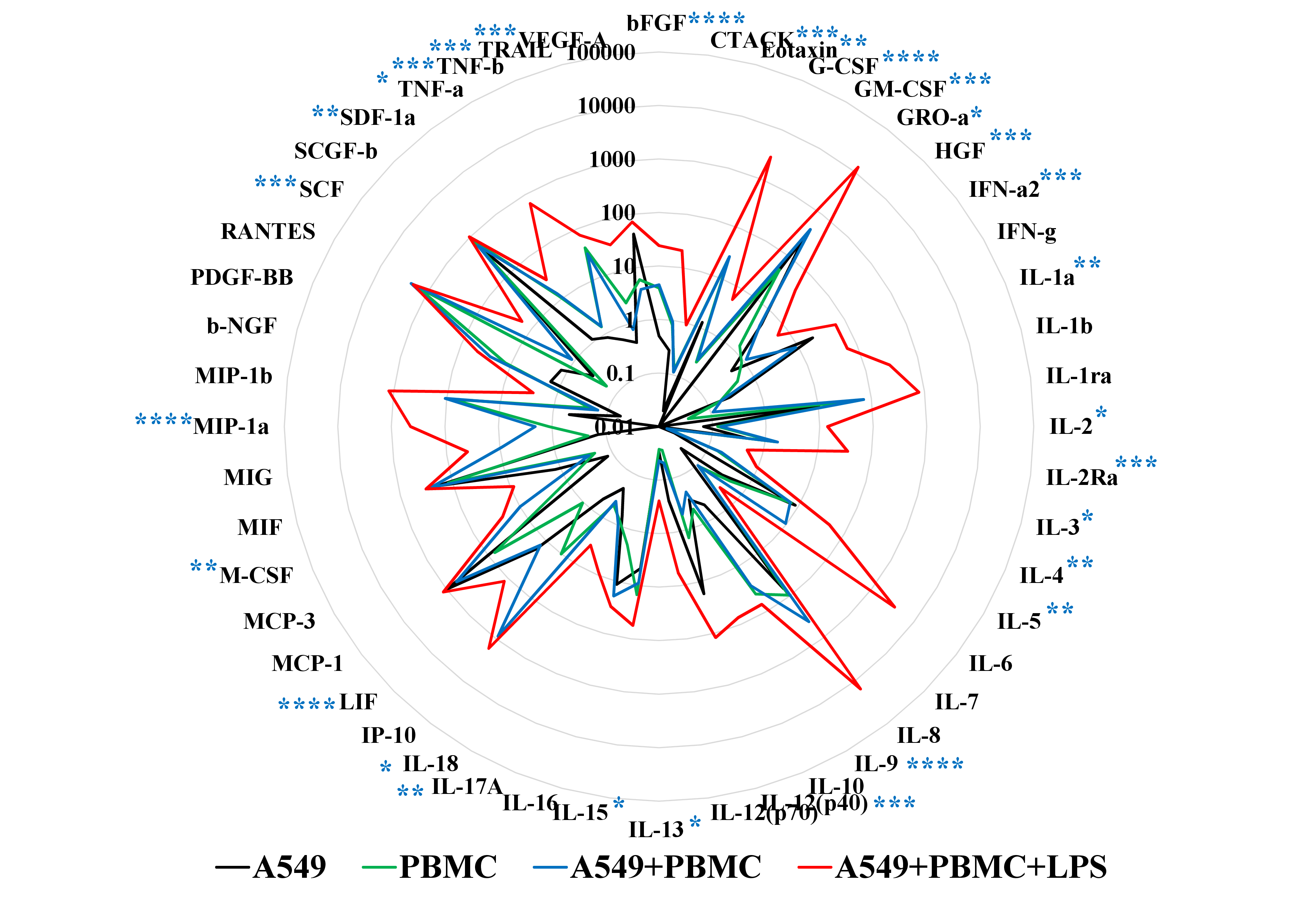

Supplement: Supplementary file 1 [file antioxidants-11-01910-s001.zip › antioxidants-1908877-Figure S2.png]
